# High-fidelity simulation versus case-based discussion for training undergraduate medical students in pediatric emergencies: a quasi-experimental study

**DOI:** 10.1016/j.jped.2024.03.007

**Published:** 2024-04-09

**Authors:** Nathalia Veiga Moliterno, Vitor Barreto Paravidino, Jaqueline Rodrigues Robaina, Fernanda Lima-Setta, Antônio José Ledo Alves da Cunha, Arnaldo Prata-Barbosa, Maria Clara de Magalhães-Barbosa

**Affiliations:** a Instituto D'Or de Pesquisa e Educação (IDOR), Departamento de Pediatria, Rio de Janeiro, RJ, Brazil; b Faculdade de Medicina de Petrópolis, Departamento de Pediatria, Petrópolis, RJ, Brazil; c Universidade do Estado do Rio de Janeiro (UERJ), Instituto de Medicina Social, Departamento de Epidemiologia, Rio de Janeiro, RJ, Brazil; d Academia Naval, Marinha do Brasil, Departamento de Educação Física e Desportos, Rio de Janeiro, RJ, Brazil; e Fundação Oswaldo Cruz, Instituto Fernandes Figueira, Unidade de Terapia Intensiva Pediátrica, Rio de Janeiro, RJ, Brazil; f Universidade Federal do Rio de Janeiro (UFRJ), Instituto de Puericultura e Pediatria Martagão Gesteira (IPPMG), Rio de Janeiro, RJ, Brazil

**Keywords:** High-fidelity simulation, Medical simulation, Pediatric emergency care, Undergraduate, Case-based discussion

## Abstract

**Objective:**

To evaluate the effect of high-fidelity simulation of pediatric emergencies compared to case-based discussion on the development of self-confidence, theoretical knowledge, clinical reasoning, communication, attitude, and leadership in undergraduate medical students.

**Methods:**

33 medical students were allocated to two teaching methods: high-fidelity simulation (HFS, *n* = 18) or case-based discussion (CBD, *n* = 15). Self-confidence and knowledge tests were applied before and after the interventions and the effect of HFS on both outcomes was estimated with mixed-effect models. An Objective Structured Clinical Examination activity was conducted after the interventions, while two independent raters used specific simulation checklists to assess clinical reasoning, communication, attitude, and leadership. The effect of HFS on these outcomes was estimated with linear and logistic regressions. The effect size was estimated with the Hedge's g.

**Results:**

Both groups had an increase in self-confidence (HFS 59.1 × 93.6, *p* < 0.001; CDB 50.5 × 88.2, *p* < 0.001) and knowledge scores over time (HFS 45.1 × 63.2, *p* = 0.001; CDB 43.5 × 56.7, p-value < 0.01), but no difference was observed between groups (group*time effect in the mixed effect models adjusted for the student ranking) for both tests (*p* = 0.6565 and *p* = 0.3331, respectively). The simulation checklist scores of the HFS group were higher than those of the CBD group, with large effect sizes in all domains (Hedges g 1.15 to 2.20).

**Conclusion:**

HFS performed better than CBD in developing clinical reasoning, communication, attitude, and leadership in undergraduate medical students in pediatric emergency care, but no significant difference was observed in self-confidence and theoretical knowledge.

## Introduction

Since the 1980s, the use of realistic simulation as a training and evaluation tool in the health area has gained significant attention and has been widely adopted. It is a teaching strategy that reproduces real situations, allowing the student to use the concepts necessary for understanding and solving problems actively.^1^^,^^2^

Realistic simulation is particularly valuable in pediatrics, as severe acute events occur infrequently. Consequently, students and residents are less exposed to training in these clinical situations.^2^, ^3^, ^4^, ^5^, ^6^ Simulation fills this gap, becoming an essential educational tool, especially in technical skills training, resuscitation, crisis management, and teamwork.^2^

The simulation tries to achieve a level of fidelity sufficient to convince users that they are involved in situations that mimic real life and can be categorized as low, medium, or high fidelity. The high-fidelity simulation incorporates a full-body computerized simulator that can be programmed to provide a realistic physiological response to students' actions.^4^^,^^7^^,^^8^

A systematic review reported that using technology-enhanced simulation for health professional education showed a consistent association with large effects on knowledge, skills, and behavior outcomes and moderate effects on patient-related outcomes.^9^ Many studies evaluating the effectiveness of high-fidelity simulation for pediatric training involve graduate and post-graduate professionals.^10^, ^11^, ^12^, ^13^, ^14^ At graduation, studies in the area of nursing predominate.^4^^,^^15^^,^^16^ Few studies evaluated high-fidelity simulation's effect on training medical students in pediatric emergencies.^17^, ^18^, ^19^, ^20^

This study aims to evaluate the effect of high-fidelity simulation training compared to case-based discussion in pediatric emergencies. Self-confidence, theoretical knowledge, and skills related to clinical reasoning, communication, attitude, and leadership in undergraduate medical students were the main variables studied.

## Methods

### Study design, setting, and population

This is a quasi-experimental study, conducted in a private medical school in Brazil. The simulation laboratory where the study was conducted has a physical area of 400 m2, with offices with one-way glass for simultaneous observation, six training rooms for pediatric, obstetric, clinical, and surgical emergencies, a home care training room, one for semiology training, and two rooms for debriefing. The Realistic Simulation in Pediatrics team is composed of eight professors (two PhDs and four MSc in Pediatrics), with extensive experience in pediatric emergencies. Thirty-three medical undergraduate internship students eligible for rotation in the pediatric emergency course during the second semester of 2020 were allocated to one of two teaching methods (interventions) according to their time availability: high-fidelity simulation training (HFS, *n* = 18) or case-based discussion (CBD, *n* = 15). The students were distributed into the two groups according to the schedule convenience of their other curricular activities.

### Ethics approval and consent to participate

This study was approved by the Research Ethics Committee (CAAE: No.83366618.1.00005245), on 03/04/2018. All students gave written informed consent.

### Study procedures

Before the start of the teaching methods, all students underwent self-confidence and theoretical knowledge tests. Then, during the first three weeks of the course, the following seven pediatric emergency topics were addressed for both groups: wheezing infants, hypovolemic shock, pneumonia/septic shock, anaphylaxis, neonatal hypoglycemia, seizures, and organophosphate poisoning All topics were based on the consensus and guidelines of the Brazilian Society of Pediatrics and the guidelines of the Pediatric Advanced Life Support program of the America Heart Association. The students were distributed into the two groups according to the schedule convenience of their other curricular activities. Group 1 was trained on a high-fidelity patient simulator (PediaSIM) in the Simulation Laboratory, and Group 2 was submitted to the CBD method. After the end of the intervention, students from both groups experienced the same self-confidence and knowledge tests applied at the beginning of the course. In addition, they were submitted to an Objective Structured Clinical Examination (OSCE)-type simulation activity in two randomly chosen scenarios among the seven topics covered in the course, all considered with the same degree of difficulty. An overview of the study procedures is presented in a flowchart in Supplement 1. Two independent raters assessed their performance in this activity with a specific checklist. Eight different teachers worked in pairs scoring the checklist.

#### High-fidelity simulation

Five to ten students participated in each simulated scenario, two of them as active players and the others as observers. The students’ roles changed with each scenario so that all students had the opportunity to be active players or observers. Three teachers participated in the simulation activity: two played the patient parents and members of the health team, and one commanded the PediaSIM responses. The training began with the case presentation, followed by the simulation of emergencies with the high-fidelity mannequin, lasting about 15 to 20 min. After that, a 40-minute debriefing took place. The students’ performances were discussed with teachers, pointing out adequate and inadequate actions and procedures. Each student participated in the simulation activity of seven different topics, and each session of HFS lasted approximately 1 hour. The total hours of HFS per student was 7 h.

#### Case-based discussion

Case-based learning (CBD) is a long-established pedagogic method that usually occurs via small group discussions of patient cases in healthcare. The CBD group discussed pediatric emergency topics in interactive activities. The same clinical scenarios were presented to the students, and they were challenged to answer on a theoretical base how to conduct anamnesis, diagnostic, and therapeutic procedures in emergencies. Each topic had outlined and structured objectives. A gamified strategy (pedagogical methodology based on games), with elements of peer-to-peer competition and teamwork was used to motivate the students through healthy competition. Adequate and inadequate responses and actions were discussed. The activity had the same duration as the simulation methodology and lasted around 60 min per theme. Each student participated in the discussion of seven topics, and each CBD lasted approximately 1 hour. The total hours of CBD per student was 7 h.

#### Assessment tools

When the idea of studying the impact of HFS training on developing clinical skills emerged, a major challenge was ensuring a robust assessment of the desired outcomes. The team of teachers devoted a lot of time judiciously reviewing and discussing the literature to develop and improve the assessment tools. Several meetings were held with experts and the teachers involved in the course until a consensus was reached on the content validity of all the clinical scenarios and the assessment instruments, as they had to contain specific items about the emergency pediatric topics addressed.

*Self-Confidence test -* The self-confidence test was a 36-item self-reported scale with 5-level Likert-type responses (0 = no confidence to 4 = full confidence) to affirmative sentences about feeling confident to provide medical care in different pediatric emergency scenarios. The total score was given by the sum of the item scores and could vary from 0 to 144 (Supplement 2A).

*Knowledge test -* The knowledge test comprised 24 multiple-choice items with specific questions about pediatric emergencies. The test result was given by the percentual of corrected answers (Supplement 2B).

*Simulation checklist* – The HFS has been used to teach pediatric emergencies to undergraduate medical students in the study medical school since 2014. The simulation checklists were already used by the Pediatrics Curricular Unit of the educational institution. They have been developed and refined over the years (since 2014). For this study, the teachers involved conducted a detailed review of the checklists based on previous experience and pediatric consensus to standardize the objectives of the different pediatric emergency topics. The simulation checklists were applied after previous training of all evaluators, showing moderate to almost perfect inter-observer and intra-observer reliability in all evaluated domains (Supplement 3A and 3B). The standardized simulation checklists were comprised of several items grouped into eight domains. Items from the domains of anamnesis, physical exam, and treatment were specific to each scenario. Items from the domains of diagnosis, systematization, communication, attitude, and leadership were common in all the scenarios (Supplement 2C). The domains of “diagnosis” and “systematization” had objective binary responses (Yes/No). The other domains were objectively scored as the percentage of correct answers to their items and subjectively scored as a 5-level Likert-type scale (very poor, poor, fair, good, and very good), depending on the rater's general impression of the student's performance in each domain. A total score was calculated as the percentage of correct answers to the items of all domains.

### Variables and data collection

The variables collected were biological sex, age, ranking order in the class (based on the student performance in medical school), the self-confidence and knowledge scores obtained before and after the interventions, and the simulation checklist scores assigned by the two evaluators after the interventions. All data were entered into Excel spreadsheets.

### Statistical analysis

Continuous variables were presented as means or medians and their measures of variation (standard deviations or interquartile ranges). Categorical variables were presented as proportions. Baseline students’ characteristics were compared between groups using the student's *t*-test or the Wilcoxon test for continuous variables and the chi-square test or Fisher's test for categorical variables.

To assess the effects of the intervention on self-confidence and knowledge, longitudinal analyses were performed using linear mixed-effect models (PROC MIXED, a procedure from the statistical software SAS OnDemand for Academics, SAS Inc., Cary, NC, USA). This analysis tests differences between groups on changes in outcomes (gains for individual students) from pre-intervention (T0) to post-intervention time (T1), accounting for correlations between the repeated measures over time and incomplete data.

The simulation checklist scores had only post-intervention measures given by two raters on two scenarios. Therefore, the average scores assigned by the two raters for each scenario (total and domain scores) in both groups were compared to assess the intervention effects on the checklist scores. Student *t*-tests for independent samples and chi-square tests were performed to compare the scores of both groups (HFS x CDB) in all dimensions. Linear and logistic regressions were also performed with the total and domain scores as dependent variables, the group as an independent variable, and student ranking as a covariate.

Effect sizes were calculated using the Hedges'g formula for continuous outcomes, with a correction for small samples, according to the “What Works Clearinghouse Procedures Handbook version 5. Hegdes'g was interpreted as follows: 0.2 (small effect size), 0.5 (medium effect size), and 0.8 (large effect size).^21^

Statistical significance was set at a two-tailed type 1 error of < 0.05 and a confidence interval of 95 %. Descriptive and regression analyses were performed using SAS OnDemand for Academics. Inter and intra-rater reliability of the simulation checklist measurements were estimated using the Stata version 9.0 (Stata Corp, College Station, Texas, USA). More details on the statistical analysis are available in the Supplement 4.

## Results

At baseline, the response rate was 97 % to the self-confidence test (one student from group 1 [HFS] did not respond) and 91 % to the knowledge test (two from group 1 and one from group 2 [CBD] did not respond). At the end of the intervention, the response rate to both tests and the checklist was 100 %.

Of the 33 students, 61 % were female, the mean age was 24, and the mean student ranking was 48.8 (for a total of 119 students in the same medical class). The mean pre-intervention scores on the self-confidence and knowledge tests were 55.1 and 44.3 %, respectively, no differences between groups were observed (Table 1).

**Table 1:** Characteristics of the participating students at the beginning of the pediatric emergency course, before the teaching interventions.

	Total	HFS	CBD	
Characteristics	*n* = 33	*n* = 18	*n* = 15	*p-value*
**Sex n (%)**				
Male Female	13 (39)	7 (39)	6 (40)	*1.000*^a^
	20 (61)	11 (61)	9 (60)	
**Age**				
mean (SD)	24.0 (1.8)	24.0 (1.4)	24.1 (2.3)	*0.9227*^b^
median (IQR)	24.0 (23.0; 25.0)	23.5 (23.0; 25.0)	24 (22; 25)	*0.6164*^c^
**Student ranking**				
mean (SD)	48.8 (27.7)	46.6 (27.7)	51.47 (28.4)	*0.6205*^b^
median (IQR)	52.0 (24.0; 75.0)	45.5 (24.0; 71.0)	55.0 (24.5; 77.0)	*0.6383*^c^
(3°, 24°] IQR	9 (27.3)	5 (28)	4 (27)	
(24°, 52°] IQR	8 (24.2)	5 (28)	3 (20)	*0.7922*^d^
(52°, 75°] IQR	8 (24.2)	5 (28)	3 (20)	
(75°, 88°] IQR	8 (24.2)	3 (17 %)	5 (33)	
Self-confidence test score*				
mean (SD)	55.1 (19.5)	59.1 (22.46)	50.5 (14.9)	*0.2052*^b^
Median	56.0 (44.3; 66.8)	66.0 (50.0;72.0)	50.0 (42.5; 59.0)	*0.1002*^c^
**Knowledge test score**⁎⁎				
mean (SD)	44.3 (9.6)	45.1 (10.2)	43.5 (9.2)	*0.6556*^b^
median (IQR)	41.7 (37.5; 52.1)	45.8 (36.5; 47.9)	39.6 (37.5; 52.1)	*0.7045*^c^

CBD, case-based discussion; HFS, high-fidelity simulation; IQR, interquartile range; SD, standard deviation.

a Chi-square test

b Student's *t*-test.

c Wilcoxon test.

d Fisher test.

⁎ Self-confidence score – the sum of 36 items scored 0 to 4 (maximum score = 144).

⁎⁎ Knowledge score – the percentage of corrected answers (maximum score = 100 %).

Descriptive statistics for each outcome are available in Supplement 4. The percentage distribution of responses for each item on the self-confidence and knowledge tests is available in Supplements 5A and 5B

**Figure 1:**
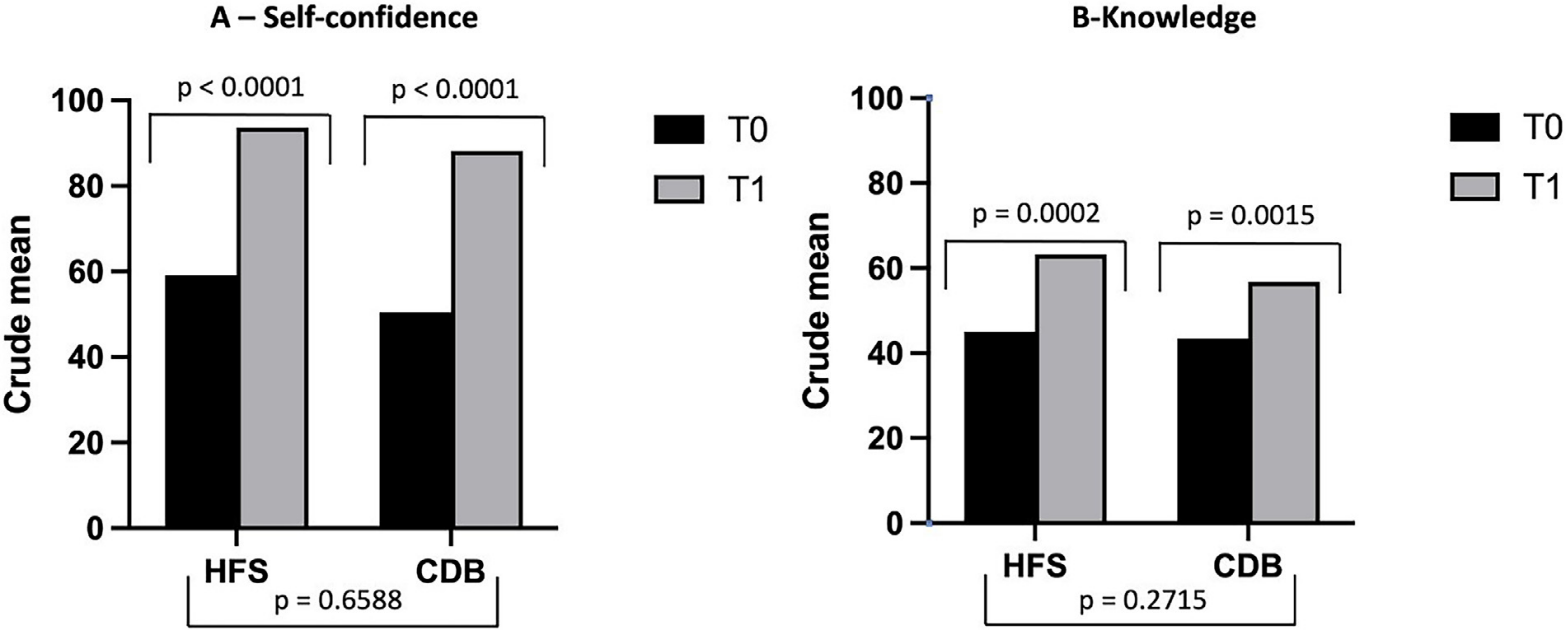
Crude means of the pre and post-intervention scores of the High-Fidelity Simulation group (HFS) and the Case-Based Discussion group (CBD) in the self-confidence test (A) and the knowledge test (B). Self-confidence scores improved significantly after intervention in both groups, without differences between the two groups (Figure 2A). Knowledge scores improved significantly after intervention in both groups, without differences between the two groups (Figure 2B).

**Figure 2:**
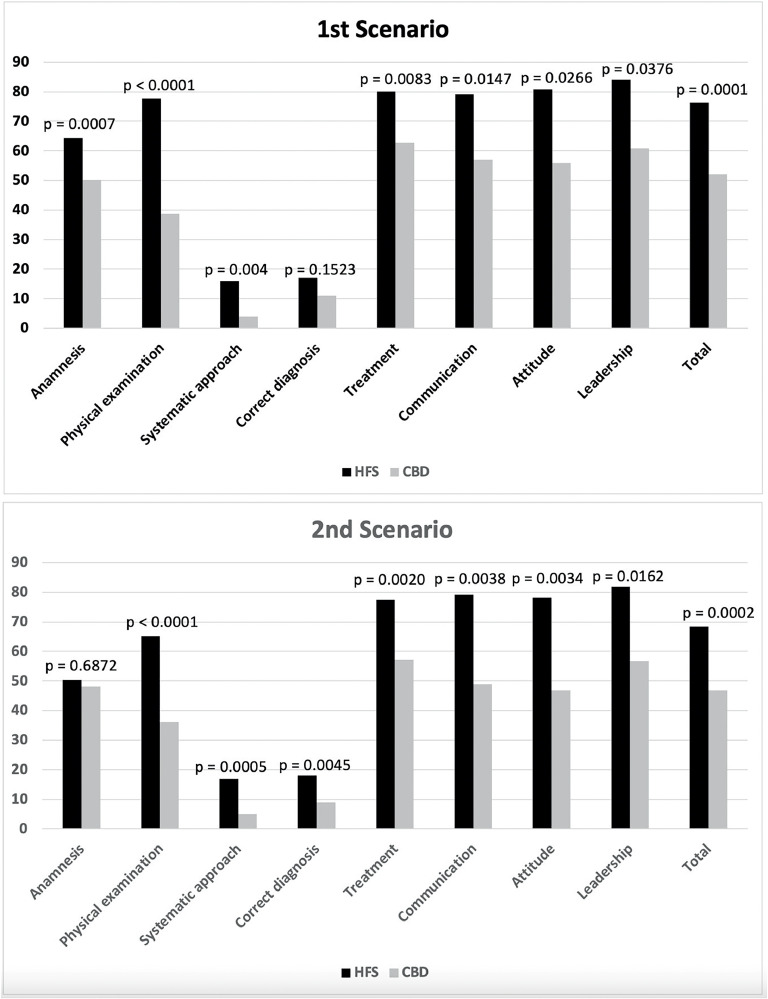
Crude means of the post-intervention scores of the High-Fidelity Simulation group (HFS) and the Case-Based Discussion group (CBD) in the simulation checklist of two scenarios.

Self-Confidence scores improved significantly after interventions in both groups (HFS 59.1 × 93.6, *p* < 0.001; CDB 50.5 × 88.2, *p* < 0.001), without differences between the two groups (*p* = 0.659) (Figure 1A). Knowledge scores improved significantly after interventions in both groups (HFS 45.1 × 63.2, *p* = 0.001; CDB 43.5 × 56.7, p-value < 0.01), without differences between the two groups (*p* = 0.272) (Figure 1B and Supplement 4). Simulation checklist post-intervention scores were significantly higher in the HFS group compared to the CBD group in both scenarios and all dimensions, except for correct diagnosis in the first scenario and anamnesis in the second scenario (Figure 2 and Supplement 4).

**Figure 3:**
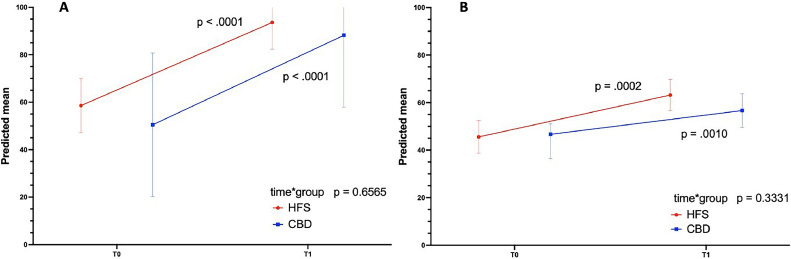
Significant increases in the predicted mean scores of the self-confidence test (A) and knowledge test (B) in the HFS group (red line) and CBD group (blue line) over time. No differences in changes over time between groups (time*group) were observed for the self-confidence and the knowledge test.

Figure 3 represents graphically the results of the mixed-effect models to test the intervention's main effect on self-confidence and knowledge outcomes adjusted for the student ranking. The time vs. group effect is the critical test of the group on score gains from pre to post-test. No differences between groups were observed regarding changes in the scores of both tests over time (*p* = 0.6565 for the self-confidence test; *p* = 0.3331 for the knowledge test). The time effect was significant for both groups in the self-confidence test (*p* < 000.1 {HFS] and *p* < 0.001 [CDB]) and in the knowledge test (*p* = 0.001 {HFS] and *p* < 0.01 ([CBD]). Table S6.1 in Supplement 6 shows the results of the mixed-effect models.

The results of linear and logistic regression models to test the effect of the HFS on the student's performance in the simulation checklist, adjusting for student ranking are available in Table S6.2 in Supplement 6. The HFS group performed significantly better than the CBD group in all dimensions with large effect sizes (Hedges g varying from 1.15 to 2.20), except in correct diagnosis in both scenarios and anamnesis in the second scenario.

## Discussion

This study evaluated the effect of high-fidelity simulation (HFS) of pediatric emergencies on different domains of knowledge, attitude, and behavior of medical internship students compared to structured case-based discussions (CBD) applied with gamified methodology. Gamification in education involves the use of game-based elements such as peer-to-peer competition, teamwork, and scoreboards to drive engagement, help students assimilate new information, and test their knowledge. This method develops an environment conducive to learning, with great student adherence, establishing itself as an active motivating methodology.^22^ The results revealed that students who participated in the HFS training performed better in clinical reasoning, communication, attitude, and leadership than those trained with CBD. Both groups showed an increase in self-confidence and theoretical knowledge scores, but there was no statistical difference between the two groups.

Self-confidence is considered a predictor of behavior in the face of emergency care, even in the case of competent physicians. Health professionals with low self-confidence in managing critically ill children can cause a severe delay in starting immediate care, leading to severe consequences for the patient.^17^ On the other hand, previous research indicated no relationship between the self-reported confidence level and students' formally assessed performance in pediatric emergency procedures.^23^ One Brazilian study showed that high fidelity simulation improves knowledge, leads the student to feel more challenged and more self-confident in recognizing the severity of the clinical case, including memory retention, and showed benefits regarding self-confidence in recognizing respiratory distress and failure in pediatric cases.^20^ In the present study, the two active methodologies increased self-confidence scores with no difference between groups. A study comparing the effect of high-fidelity versus medium-fidelity simulation in pediatrics revealed that medical students improved self-confidence scores with both methods. HFS was superior in the knowledge of Pediatric Advanced Life Support (PALS) algorithms compared to simulation on traditional low-fidelity (non-computerized) mannequins.^17^ Coolen et al. compared three training methods for acute pediatric emergencies – high-fidelity video-assisted real-time simulation (VARS), problem-based learning (PBL), and Pediatric Advanced Life Support (PALS). Although the authors found no statistical differences in the self-confidence scores between groups, they observed a slightly lesser increase in the VARS group compared to the other groups.^10^ They argue that the stress associated with real-time actions could help recognize the difficulty of conducting a structured approach during stressful circumstances.

Both groups showed an increase in the theoretical knowledge test scores, with no difference between groups. Literature findings are divergent. One study revealed that the simulation of intensive care topics resulted in higher scores on multiple-choice tests for knowledge evaluation and was considered more enjoyable than lectures by final-year medical students.^24^ Couto et al. found results similar to ours when comparing HFS with CBD for teaching pediatric emergencies to medical students.^14^ No difference was observed regarding acquiring and retaining knowledge, but HFS was superior in terms of student satisfaction. On the other hand, according to a study by Avabratha et al. with final-year medical students, both lectures and high-fidelity simulation improved learning outcomes. However, knowledge scores were significantly higher after lectures than simulation.^25^ Finally, a study by Besbes et al. showed that both HFS and video-based training are effective educational strategies for septic shock training of internship students, with HFS appearing to be superior in short-term knowledge retention.^26^

In the present study, the HFS proved superior to CBD in assessing student performance through simulation checklists. A point that draws attention and corroborates the role of simulation is that only the items that evaluated the correctness of the diagnosis (in the first scenario) and the anamnesis (in the second scenario) exhibited no difference between the groups. From the authors’ point of view, these items depend more on theoretical knowledge about the topics addressed than on practical skills and attitudes, which are the pillars of simulated activities. Indeed, when comparing HSF with CBD in the present study, the effect sizes of HSF training on communication, attitude, and leadership were large. The present findings support the need for training technical and non-technical skills related to behavior, attitude, leadership, and communication during undergraduate degrees. Another study revealed that interns who participate in pediatric traumatic brain injury training with HFS compared to clinical case discussion better understood, and applied pre-established rules for traumatic brain injury, and retained them longer.^18^

### Limitations

This study has limitations. The main one, imposed by the COVID-19 pandemic, was the sample size. The plan was to include the 119 students who would rotate in the pediatric emergency course during 2020 based on a sample size calculation. The non-significant statistics of this study may be due to a lack of power. Another limitation is the non-randomized design. The students were distributed into the two groups according to the schedule convenience of their other curricular activities. Despite this, demographic characteristics and pre-intervention scores were similar in both groups. In addition, the authors used the covariate “student ranking” to adjust the regression models. The use of variable pairs of scenarios in the final assessment can also be pointed out as a limitation. It was a necessary strategy to avoid prior knowledge of the topics by the students, given the impossibility of evaluating all students on the same day. However, the scenarios were carefully designed with similar degrees of difficulty by the team of teachers. Finally, the study was conducted in a single educational institution with a specific physical structure and human resources, limiting its generalization to other institutions with different characteristics.

Despite these limitations, the results of this study corroborated with the empirical perception that HFS in pediatrics is necessary to improve the technical and non-technical skills of undergraduate medical students. The positive impact of this strategy resulted in the expansion and earlier introduction of the method in the curriculum. Currently, it has been inserted since the pre-internship in pediatrics. The present study contributes evidence on the positive effect of using high-fidelity simulation on the acquisition of competencies, skills, and attitudes in undergraduate students in pediatric emergency settings.

## Conflicts of interest

The authors declare no conflicts of interest.

## Appendix Supplementary materials

Image, application 1

Image, application 2

Image, application 3

Image, application 4

Image, application 5

Image, application 6

Image, application 7

Image, application 8

Image, application 9

Image, application 10
