# Supplementary material for: High-fidelity simulation versus case-based discussion for training undergraduate medical students in pediatric emergencies: a quasi-experimental study
Source: J Pediatr (Rio J). 2024 Apr 9;100(4):422–9. doi: 10.1016/j.jped.2024.03.007 (PMC11331236; doi:10.1016/j.jped.2024.03.007)
Supplement: Supplementary file 2 [file mmc2.docx]

**High-fidelity simulation versus case-based discussion for training undergraduate medical students in pediatric emergencies: a quasi-experimental study.**

Nathalia Veiga Moliterno, Vitor Barreto Paravidino, Jaqueline Rodrigues Robaina, Fernanda Lima-Setta, Antônio José Ledo Alves da Cunha, Arnaldo Prata-Barbosa and Maria Clara de Magalhães-Barbosa.


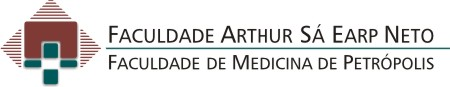


Student:_____________________________________________date:___/___/___

On a scale of **0 to 4**, with **0 being the lack of confidence** and **4 being complete confidence**, categorize your level of confidence in addressing the following questions:

| How confident are you that you will be able to: | 0 | 1 | 2 | 3 | 4 |
| --- | --- | --- | --- | --- | --- |
| 1. collect an adequate targeted anamnesis from a paediatric critical patient |  |  |  |  |  |
| 1. communicate clearly with relatives of a paediatric critical patient |  |  |  |  |  |
| 1. welcome the patient and family during paediatric emergency care |  |  |  |  |  |
| 1. carry out clear communication in a closed loop with the assistance team during emergency care |  |  |  |  |  |
| 1. carry out teamwork effectively in paediatric emergency care |  |  |  |  |  |
| 1. prioritize actions in a pediatric emergency care |  |  |  |  |  |
| 1. identify the actions related to each ABCDE approach phase |  |  |  |  |  |
| 1. prescribe medications of common use in pediatrics correctly (indication, route, and dose) |  |  |  |  |  |
| 1. recognize signs and symptoms of pediatric respiratory failure |  |  |  |  |  |
| 1. make an appropriate choice in the type of device for oxygen therapy in childhood |  |  |  |  |  |
| 1. appropriately intervene in a respiratory failure in a child |  |  |  |  |  |
| 1. assess the effectiveness of your interventions in addressing respiratory failure |  |  |  |  |  |
| 1. perform proper treatment of a child with upper airway obstruction? |  |  |  |  |  |
| 1. perform proper treatment of a child with lower airway obstruction? |  |  |  |  |  |
| 1. perform adequate clinical management of severe asthma in pediatrics in the emergency room |  |  |  |  |  |
| 1. recognize signs and symptoms of shock in pediatrics |  |  |  |  |  |
| 1. classify shock in pediatrics as to its etiology |  |  |  |  |  |
| 1. make an appropriate intervention in a pediatrics shock |  |  |  |  |  |
| 1. adequately approach the first hour of septic shock in a pediatric patient |  |  |  |  |  |
| 1. evaluate the effectiveness of your interventions in the initial approach to shock in pediatrics |  |  |  |  |  |
| 1. recognize signs and symptoms of an acute neurological event in pediatrics |  |  |  |  |  |
| 1. appropriately intervene in a seizure in a child |  |  |  |  |  |
| 1. identify risk factors for neonatal hypoglycaemia |  |  |  |  |  |
| 1. recognise signs and symptoms of a newborn with neonatal hypoglycaemia |  |  |  |  |  |
| 1. appropriately intervene in a case of a newborn with neonatal hypoglycemia |  |  |  |  |  |
| 1. recognise signs and symptoms of symptomatic hypoglycaemia in a child/schoolchild |  |  |  |  |  |
| 1. appropriately intervene in a case of a child/schoolchild with symptomatic hypoglycaemia |  |  |  |  |  |
| 1. diagnose patients involved in a poisonous animal accident |  |  |  |  |  |
| 1. classify as to the severity of the child who suffered an ophidian accident |  |  |  |  |  |
| 1. make the initial addressing of a child with ophidian accident clinical signs |  |  |  |  |  |
| 1. recognize signs and symptoms of anaphylaxis in pediatrics |  |  |  |  |  |
| 1. perform adequate treatment of anaphylaxis in pediatrics |  |  |  |  |  |
| 1. recognize signs and symptoms of acute bacterial meningitis in pediatrics |  |  |  |  |  |
| 1. perform appropriate diagnostic and therapeutic approach of pediatric patient with acute meningitis |  |  |  |  |  |
| 1. recognize signs of severe condition in an adult patient |  |  |  |  |  |
| 1. make the initial approach of an acutely ill adult patient |  |  |  |  |  |
