# Supplementary material for: High-fidelity simulation versus case-based discussion for training undergraduate medical students in pediatric emergencies: a quasi-experimental study
Source: J Pediatr (Rio J). 2024 Apr 9;100(4):422–9. doi: 10.1016/j.jped.2024.03.007 (PMC11331236; doi:10.1016/j.jped.2024.03.007)
Supplement: Supplementary file 3 [file mmc3.docx]

**High-fidelity simulation versus case-based discussion for training undergraduate medical students in pediatric emergencies: a quasi-experimental study.**

Nathalia Veiga Moliterno, Vitor Barreto Paravidino, Jaqueline Rodrigues Robaina, Fernanda Lima-Setta, Antônio José Ledo Alves da Cunha, Arnaldo Prata-Barbosa and Maria Clara de Magalhães-Barbosa.

THEORIC TEST- REALISTIC SIMULATION IN PEDIATRICS

Student: ___________________________________________________date:___/___/___

1. In pediatric emergency situations, facing an unconscious (unresponsive) **infant**, which of the pulses described below are the ones of choice for verification if the patient is in cardiac arrest?
2. Radial and brachial
3. Carotid and femoral
4. Femoral and brachial
5. Brachial and carotid
6. In pediatric emergency situations, facing an unconscious (unresponsive) infant, which of the pulses described below are the ones of choice for verification if the patient is in cardiac arrest?
7. Radial and brachial
8. Carotid and femoral
9. Femoral and brachial
10. Brachial and carotid
11. In pediatric patients, the main cause of cardiac arrest is due to:
12. Hypothermia
13. trauma
14. Respiratory failure
15. Sepsis
16. A 20kg child has fever, irritability, cold extremities and prolonged hair filling time. The appropriate initial fluid for bolus administration for this child is:
17. 100 ml of isotonic saline for 30 to 60 min
18. 200 ml of SG5% in 20 minutes
19. 400 ml of isotonic saline or lactated Ringer's in 20 minutes
20. 800 ml of isotonic saline or lactated Ringer's in 20 minutes
21. A 3-year-old child arrives at the emergency room with a low level of consciousness, pale, central cyanosis 1+/4+, thin wrists, moderate respiratory effort. On the basis of this initial situation, its first intervention should be:
22. Establish peripheral vascular access
23. Provide oxygen under mask
24. Release airway
25. Perform volume expansion

Student: ___________________________________________________date:___/___/___

1. The inhaled dose of epinephrine for infants or children is:
2. 0.1ml/kg of 1:1,000 solution
3. 0.1ml/kg of 1: 10,000 solution
4. 0.5ml/kg of 1:1,000 solution
5. 0.5ml/kg of 1:10,000 solution
6. A previously healthy 5-month-old infant was admitted to the cyanotic emergency room, with red spots throughout the body, irritability, and respiratory distress beginning in 30 minutes. The grandmother who brought the child. She claims to have offered infant formula today for the first time. **On physical examination of admission:** Agitated, pale, spots on the body, slow PCP, fine and fast peripheral pulses. Tachypneic, moderate respiratory effort, MVUA, bilateral wheezing. Erythematous papules on the trunk, face and limbs. Absence of meningeal signs. HR 195 FR: 50 SatO2: 88% Tax 36°C PA 90X55mmHg (normal for age).

Given the situation presented, what is the primary conduct for solving the case?

1. Vascular access and volume expansion
2. Intramuscular adrenaline application
3. Inhaled salbutamol
4. Systemic corticosteroid therapy
5. The inhaled dose of epinephrine for infants or children is:
6. 0.1mg/kg (0.1ml/kg)
7. 0.01mg/kg (0.1ml/kg)
8. 0.3mg/kg (0.3ml/kg)
9. 0.03mg/kg (0.3ml/kg) of 1:10,000 solution
10. A 15-month-old infant, weighing 13 kg, presented hyaline coryza, dry cough and fever of 38ºC this evening. He was treated with paracetamol, with a good response, and fell asleep. After 4 hours of sleep, he started an intense episode of barking cough, hoarseness and progressive dyspnea. On examination, he presented stridor at rest, cyanosis of the extremities and subcostal circulation. His parents immediately took him to the Emergency Care Unit. What specific therapeutic conduct should be initiated in this case?
11. Adrenaline 6.5 ml inhalation
12. Salbutamol 4 jets in the aerocamera
13. Salbutamol 2 jets in the aerocamera
14. Adrenaline 5 ml inhalation
15. The first clinical manifestation of shock in pediatrics is:
16. Oliguria
17. Hypotension
18. Tachycardia
19. Reduction of peripheral pulses

Student: ___________________________________________________date:___/___/___

1. Among the situations below, in which of them could you perform lumbar puncture?
   1. Patient with hemodynamic instability
   2. Patient with anisocoria
   3. Total platelet count of 80,000
   4. Infected scabies in the lumbosacral region
2. A 10-year-old student weighing 35 kg, diagnosed with diabetes mellitus for 10 months, was on NPH insulin in the morning and in the evening before meals. He arrived at the emergency room at 10: 00 a.m. with a history of fainting, sweating and tremors at school. When asked by the emergency physician, the mother mentioned that the minor did not want to have coffee before going to school. Intake capillary blood glucose 30 mg/dl. What is the correct treatment for this clinical situation?
3. SG5% - 2 to 4 ml/Kg
4. SG10% - 2 to 4 ml/Kg
5. SG25% - 2 to 4 ml/Kg
6. SG50% - 2 to 4 ml/Kg
7. A 15-month-old infant was admitted to the Emergency Care Unit unconscious presenting conjugate deviation of the look up, spasticity of the limbs and sialorrhea. The mother reports that the painting started 20 minutes ago. Estimated weight of 10 kg. After the initial critical patient approach, which of the following indicates the correct conduct to be initiated?
8. Diazepan 0.6ml intranasal
9. Intravenous midazolam 2ml
10. Midazolan 2ml intranasal
11. Diazepan 0.6ml intravenous
12. The results found in the cerebrospinal fluid suggesting bacterial meningitis are
    a) pleocytosis with a predominance of polymorphonuclear, hypoproteinorachia and hyperglycorrhachiab
    b) pleocytosis with a predominance of mononucleraes, hyperproteinorachia andnormoglycorrhachiac
    c) pleocytosis with a predominance of polymorphonuclear, hyperproteinorachia and hypoglycorrhachiaad
    d) pleocytosis with a predominance of mononucleraes, hypoproteinorachia and hypoglycorrhachia
13. Faced with a preschooler with decompensated shock, what is the vascular access of choice for infusion of fluid therapy?
14. Central venous access
15. Peripheral venous access
16. Peripheral central infusion catheter
17. Intraosseous access

Student: ___________________________________________________date:___/___/___

1. The intervention that has a direct effect on reducing mortality in anaphylaxis is:
   1. Systemic corticosteroid
   2. Adrenaline
   3. Antihistamine
   4. Expansion with crystalloid
2. NB with 48 hours of life, premature at 35 weeks and 5 days, due to pre-eclampsia, PN 2450g. She is staying with her exclusively breastfed mother. Mother says that "he is lazy", nurse noticed that he is presenting tremors in limbs and sweating and immediately called the pediatrician on duty, who performed capillary blood glucose (23 mg/dl). What is the most appropriate conduct?
   1. Offer formula in the cup and observe
   2. Make 25% glucose flush - 2 ml/kg venous and then offer breast
   3. Make glucose flush 10 % - 2 ml/kg venous and then offer breast
   4. Make 10% glucose flush 2 ml/kg venous and then start venous hydration
3. A 4-year-old child was admitted to the emergency room with coughing, tiredness and “wheezing in the chest”. On examination, the patient was found to have dyspnea with a notch and a wing beat of the nose, Sato2 88%, respiratory auscultation with diffuse wheezing.

The treatment sequence after initiation of inhaled oxygen therapy should be:
a) B2 short-term ( inhaled) agonist

b) Long-lasting (inhaled) B2 agonist
c) Venous Magnesium Sulfate

d) Adrenaline (inhaled)

1. What is the maximum dose of adrenaline and minimum interval for subsequent dose, if necessary, for treatment of anaphylactic shock?
   1. 0.01 mg/kg – 5 minute interval
   2. 0.1mg/kg – 5-minute interval
   3. 0.05mg/kg – 15-minute interval
   4. 0.5mg/kg – 15-minute interval
2. Among the actions that are a priority in the approach of pediatric patients in the **first hour** of septic shock are, except:
   1. Obtaining deep venous access
   2. Collection of cultures and Measurement of lactate
   3. Beginning of empirical broad-spectrum antibiotic therapy
   4. Volume Expansion

Student: ___________________________________________________date:___/___/___

1. A 9-month-old male patient was admitted to the emergency room with diffuse wheezing and moderate respiratory distress (tachypnea and subcostal circulation). Mother reports recurrent episodes of wheezing associated with “viruses”. In the last three months it was three times the emergency for the same reason. Weight 10kg - Sato2 90%. The medication and dose indicated for rescue:
   a) Nebulization with 10 drops of Fenoterol + 3ml saline 0.9% every 2 hours

b) ) Nebulization with 10 drops of Fenoterol + 3ml saline 0.9% every 20 minutes in the first hour
c) salbutamol spray with spacer : 3 jets every 20 minutes in the first hour
d) Salbutamol spray with spacer : 3 jets every 2 hours

1. In cases of ophidian accident, we base the calculation of the amount of ampoules of antivenom to be administered:
   1. In the patient's body weight
   2. On the patient's body surface
   3. In the classification of the severity of the accident
   4. In the species of the poisonous animal involved
2. In cases of botropic accident, we **must** NOT:
   1. Perform local cleaning, lifting of the limb and removal of adornments
   2. Start empirical antibiotic therapy
   3. Hydrate and maintain diuresis greater than 1 ml/kg/h
   4. Administer pain relieving painkillers
3. A 4-year-old boy underwent lumbar puncture and the cerebrospinal fluid results showed a cloudy cerebrospinal fluid, cellularity of 1000 cells/mm3 with a predominance of polymorphonuclear cells (90%); proteinorrhachia of 250mg/dL, glycorrhachia of 23mg/dL (glycemia 80mg/dL) . Bacterioscopy showed numerous Gram positive coconuts in pairs and chains. What is the most likely etiologic agent for meningitis described above?
   a) pneumococcus

b) hemophilus

c) staphylococcus

d) meningococcus

THEORIC TEST – REALISTIC SIMULATION

Student: ___________________________________________________date:___/___/___

Answers

| 1 | A | B | C | D |
| --- | --- | --- | --- | --- |
| 2 | A | B | C | D |
| 3 | A | B | C | D |
| 4 | A | B | C | D |
| 5 | A | B | C | D |
| 6 | A | B | C | D |
| 7 | A | B | C | D |
| 8 | A | B | C | D |
| 9 | A | B | C | D |
| 10 | A | B | C | D |
| 11 | A | B | C | D |
| 12 | A | B | C | D |
| 13 | A | B | C | D |
| 14 | A | B | C | D |
| 15 | A | B | C | D |
| 16 | A | B | C | D |
| 17 | A | B | C | D |
| 18 | A | B | C | D |
| 19 | A | B | C | D |
| 20 | A | B | C | D |
| 21 | A | B | C | D |
| 22 | A | B | C | D |
| 23 | A | B | C | D |
| 24 | A | B | C | D |
| 25 | A | B | C | D |
