# Supplementary material for: High-fidelity simulation versus case-based discussion for training undergraduate medical students in pediatric emergencies: a quasi-experimental study
Source: J Pediatr (Rio J). 2024 Apr 9;100(4):422–9. doi: 10.1016/j.jped.2024.03.007 (PMC11331236; doi:10.1016/j.jped.2024.03.007)
Supplement: Supplementary file 4 [file mmc4.docx]

**High-fidelity simulation versus case-based discussion for training undergraduate medical students in pediatric emergencies: a quasi-experimental study.**

Nathalia Veiga Moliterno, Vitor Barreto Paravidino, Jaqueline Rodrigues Robaina, Fernanda Lima-Setta, Antônio José Ledo Alves da Cunha, Arnaldo Prata-Barbosa and Maria Clara de Magalhães-Barbosa.


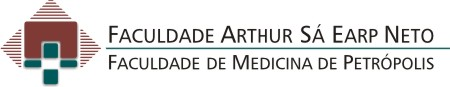


GLOBAL ASSESSMENT SCALE: WHEEZING INFANT SCENARIO

Student: ________________________________________ Date:___/___/___

How many scenarios have you participated or watched?________

Have you ever been on this case?________ Have you worked on this case?__________

□ member team □ leader team

Evaluator:_____________________________________________________________

BRIEFING

| Clothing (NR32)  Attention to guidelines  Posture |
| --- |

| Very poor | Bad | Regular | Good | Very good |
| --- | --- | --- | --- | --- |

1. ANAMNESIS (Number of hits:______/14)

| 10 months – weight 10kg - began 12 hours ago with dry cough and tiredness.  History of bronchiolitis with four months of age. In the last six months parents led her five times to the emergency room with a condition similar to the current one.  Triggering factor: there is a work at home making a lot of dust.  Does not regularly follow up with a pediatrician.  Does not use drugs in a regular basis.  Updated vaccination card |
| --- |

| Did the student ask? | | | | YES | | NO |
| --- | --- | --- | --- | --- | --- | --- |
| Child's age | | | |  | |  |
| Child's weight | | | |  | |  |
| Allergies | | | |  | |  |
| Last Feeding | | | |  | |  |
| Onset of clinical picture | | | |  | |  |
| Previous diseases/underlying disease | | | |  | |  |
| Previous episodes/Previous hospitalizations | | | |  | |  |
| Use of medications | | | |  | |  |
| Fever | | | |  | |  |
| Contact with sick people/ someone else in the family with the same symptoms | | | |  | |  |
| Triggering factors *(work at home) | | | |  | |  |
| Respiratory symptoms | | | |  | |  |
| Asked about the child's current general condition | | | |  | |  |
| Family history of asthma | | | |  | |  |
| Very poor | Bad | Regular | Good | | Very good | |

1. PHYSICAL EXAMINATION (No. of hits _____/14)

| PHYSICAL EXAMINATION | YES | NO |
| --- | --- | --- |
| A) Airway |  |  |
| B) Breathing |  |  |
| Respiratory rate |  |  |
| Presence of respiratory effort |  |  |
| Respiratory auscultation |  |  |
| Presence of central cyanosis |  |  |
| C Cardiovascular |  |  |
| Pulse Range |  |  |
| Heart rate |  |  |
| Blood pressure |  |  |
| Cappilary filling time |  |  |
| Signs of dehydration | Does not apply |  |
| D |  |  |
| Level of consciousness |  |  |
| Pupillary evaluation | Does not apply |  |
| Asked for abnormal tone/movements | Does not apply |  |
| Fontanels | Does not apply |  |
| Meningeal signs | Does not apply |  |
| AND EXPOSURE |  |  |
| Patient exposure |  |  |
| Body temperature |  |  |
| Skin injuries |  |  |
| Abdominal exam |  |  |

Did the student get the correct diagnosis? ( ) yes ( ) no

1. THERAPEUTIC ACTIONS (No. of hits: _________/14)

|  | | | | | YES | | NO |
| --- | --- | --- | --- | --- | --- | --- | --- |
| Monitoring | | | | |  | |  |
| Raised the head of the bed | | | | |  | |  |
| Installed oxygen delivery | | | | |  | |  |
| Oxygen correct liters/min and correct device | | | | |  | |  |
| Requested bronchodilator (salbutamol spray/fenoterol nebulization) | | | | |  | |  |
| Appropriate dose of bronchodilator | | | | |  | |  |
| Proper administration of bronchodilator (nebulizer or spacer) | | | | |  | |  |
| Requested venous access (before requesting medication) | | | | |  | |  |
| Administration of systemic corticosteroids | | | | |  | |  |
| Correct dose of corticosteroid in mL | | | | |  | |  |
| Correct corticosteroid pathway | | | | |  | |  |
| Re-evaluated the patient | | | | |  | |  |
| Indicated bronchodilator repetition with adequate interval | | | | |  | |  |
| Discussed hospitalization | | | | |  | |  |
| Very poor | Bad | Regular | Good | | Very good | |  |

Performed therapeutic actions in a systematic manner/in the order ( )YES ( ) NO

What is the diagnosis made by the student? ____________________________________________

1. COMMUNICATION (Number of hits: ____/10)

|  | Yes | No |
| --- | --- | --- |
| Introduced himself to the family |  |  |
| Qualified himself |  |  |
| Asked the mother's name |  |  |
| Asked the child's name |  |  |
| Appropriate non-verbal communication (posture) |  |  |
| Performs communication with team in closed loop in critical/intervention moments |  |  |
| Speaks in a calm and normal tone of voice |  |  |
| Explains the clinical situation to the patient/family member clearly and technically appropriate at the end of the treatment |  |  |
| Explains the clinical situation to the patient/family empathetically |  |  |
| Clarifies the steps to be taken next |  |  |

| Very poor | Bad | Regular | Good | Very good |
| --- | --- | --- | --- | --- |

1. PROFESSIONAL BEHAVIOR/ATTITUDE (No. of hits:____/7)

|  | | | | | | YES | | NO |
| --- | --- | --- | --- | --- | --- | --- | --- | --- |
| Works well in a team | | | | | |  | |  |
| Concomitance of actions | | | | | |  | |  |
| Systematizes care | | | | | |  | |  |
| Has ethical behavior throughout the service | | | | | |  | |  |
| Demonstrated self-confidence throughout the training | | | | | |  | |  |
| The assistance had fluidity (adequate timing, agility) | | | | | |  | |  |
| Made decisions aimed at the appropriate use, effectiveness and cost-effectiveness of the workforce, medicines, equipment, procedures and practices – that is: DID NOT PERFORM UNNECESSARY CONDUCT (UNNECESSARY examinations and procedures) | | | | | |  | |  |
| Very poor | Bad | Regular | Good | Very good | |  |  |  |

1. LEADERSHIP (No. of hits:_____/4)

|  | YES | NO |
| --- | --- | --- |
| Had commitment with the patient |  |  |
| Had an empathic attitude |  |  |
| Showed the ability to make decisions |  |  |
| Managed care appropriately |  |  |

| Very poor | Bad | Regular | Good | Very good |
| --- | --- | --- | --- | --- |

1. Debriefing

| Participation |
| --- |

| Very poor | Bad | Regular | Good | Very good |
| --- | --- | --- | --- | --- |

1. OVERALL PERFORMANCE **(RATER IMPRESSION**)

| Very poor | Bad | Regular | Good | Very good |
| --- | --- | --- | --- | --- |

1. OBJECTIVE ASSESSMENT - TOTAL NUMBER OF HITS______/63

Notes:

Scenario duration: ______________________Intercurrences during the scenario: __________ __________________________________________________________________________________________________________________________________________________________

Debriefing Duration:__________________ Debriefing complications: _____________________

_____________________________________________________________________________

Mark YES or NO on the checklist

At the end of the checklist – note the total number of hits

Mark VERY POOR, BAD, REGULAR, GOOD and VERY GOOD – below each dimension according to your impression and also your general impression of the service
