# Supplementary material for: High-fidelity simulation versus case-based discussion for training undergraduate medical students in pediatric emergencies: a quasi-experimental study
Source: J Pediatr (Rio J). 2024 Apr 9;100(4):422–9. doi: 10.1016/j.jped.2024.03.007 (PMC11331236; doi:10.1016/j.jped.2024.03.007)
Supplement: Supplementary file 5 [file mmc5.docx]

**High-fidelity simulation versus case-based discussion for training undergraduate medical students in pediatric emergencies: a quasi-experimental study.**

Nathalia Veiga Moliterno, Vitor Barreto Paravidino, Jaqueline Rodrigues Robaina, Fernanda Lima-Setta, Antônio José Ledo Alves da Cunha, Arnaldo Prata-Barbosa and Maria Clara de Magalhães-Barbosa.

**Table S4.** Inter-observer reliability of objective and subjective measures of the two scenarios of the OSCE checklist assigned by two observers

| **Checklist domains** | **INTER OBSERVER RELIABILITY** | |
| --- | --- | --- |
|  | Quadratic weighted kappa – kw^2^ (95% CI) | |
|  | **Subjective** | **Objective** |
| **Anamnesis*** |  |  |
| 1^st^ scenario | 0.716 (0.612 - 0.770) | 0.712 (0.694 - 0.798) |
| 2^nd^ scenario | 0.741 (0.727 - 0.791) | 0.739 (0.676 - 0.805) |
| **Physical exam*** |  |  |
| 1^st^ scenario | 0.927 (0.866 - 0.936) | 0.952 (0.921 - 0.977) |
| 2^nd^ scenario | 0.758 (0.720 - 0.810) | 0.720 (0.644 - 0.816) |
| **Diagnosis¶** |  |  |
| 1^st^ scenario | - | 0.871 (0.624 - 1.000) |
| 2^nd^ scenario | - | 1.000 |
| **Treatment** |  |  |
| 1^st^ scenario | 0.830 (0.784 - 0.834) | 0.746 (0.339 - 0.835) |
| 2^nd^ scenario | 0.812 (0.617 - 1.000) | 0.928 (0.830 - 0.969) |
| **Communication** |  |  |
| 1^st^ scenario | 0.875 (0.835 - 0.882) | 0.833 (0.789 - 0.977) |
| 2^nd^ scenario | 0.855 (0.647 - 0.872) | 0.928 (0.846 - 0.945) |
| **Systematization¶** |  |  |
| 1^st^ scenario | - | 0.867 (0.691 - 1.000) |
| 2^nd^ scenario | - | 0.874 (0.705 - 1.000) |
| **Attitude*** |  |  |
| 1^st^ scenario | 0.792 (0.622 - 0.861) | 0.710 (0.536 - 0.854) |
| 2^nd^ scenario | 0.823 (0.715 - 0.872) | 0.857 (0.743 - 0.942) |
| **Leadership*** |  |  |
| 1^st^ scenario | 0.804 (0.737 - 0.901) | 0.770 (0.734 - 0.931) |
| 2^nd^ scenario | 0.769 (0.639 - 0.882) | 0.793 (0.726 - 0.874) |
| **Total*** |  |  |
| 1^st^ scenario | 0.813 (0.564 - 0.863) | 0.910 (0.867 - 0.956) |
| 2^nd^ scenario | 0.889 (0.842 - 0.928) | 0.843 (0.733 - 0.932) |

*quadratic wieghted kappa for ordinal variables

¶ simple kappa for binary variables
