# Supplementary material for: High-fidelity simulation versus case-based discussion for training undergraduate medical students in pediatric emergencies: a quasi-experimental study
Source: J Pediatr (Rio J). 2024 Apr 9;100(4):422–9. doi: 10.1016/j.jped.2024.03.007 (PMC11331236; doi:10.1016/j.jped.2024.03.007)
Supplement: Supplementary file 6 [file mmc6.docx]

**High-fidelity simulation versus case-based discussion for training undergraduate medical students in pediatric emergencies: a quasi-experimental study.**

Nathalia Veiga Moliterno, Vitor Barreto Paravidino, Jaqueline Rodrigues Robaina, Fernanda Lima-Setta, Antônio José Ledo Alves da Cunha, Arnaldo Prata-Barbosa and Maria Clara de Magalhães-Barbosa.

**Table S5.** Intra-observer reliability between the objective and subjective measures of the two scenarios of the OSCE checklist assigned by each one of two observers.

| **Checklist domains** | **INTRA OBSERVER RELIABILITY** | |
| --- | --- | --- |
|  | Quadratic weighted kappa – kw^2^ (I95% CI) | |
|  | Observer 1 | Observer 2 |
| **Anamnesis*** |  |  |
| 1^st^ scenario | 0.629 (0.501 - 0.740) | 0.655 (0.477 - 0.797) |
| 2^nd^ scenario | 0.566 (0.332 - 0.581) | 0.667 (0.560 - 0.702) |
| **Physical exam*** |  |  |
| 1^st^ scenario | 0.907 (0.816 - 0.949) | 0.925 (0.851 - 0.960) |
| 2^nd^ scenario | 0.824 (0.780 - 0.863) | 0.757 (0.575 - 0.797) |
| **Diagnosis¶** |  |  |
| 1^st^ scenario | - | - |
| 2^nd^ scenario | - | - |
| **Treatment** |  |  |
| 1^st^ scenario | 0.835 (0.746 - 0.911) | 0.676 (0.524 - 0.763) |
| 2^nd^ scenario | 0.834 (0.762 - 0.849) | 0.677 (0.383 - 0.799) |
| **Communication** |  |  |
| 1^st^ scenario | 0.895 (0.865 - 0.929) | 0.727 (0.486 - 0.863) |
| 2^nd^ scenario | 0.911 (0.834 - 0.934) | 0.926 (0.922 - 0.934) |
| **Systematization¶** |  |  |
| 1^st^ scenario | - | - |
| 2^nd^ scenario | - | - |
| **Attitude*** |  |  |
| 1^st^ scenario | 0.958 (0.906 - 0.985) | 0.932 (0.920 - 0.966) |
| 2^nd^ scenario | 0.923 (0.902 - 0.942) | 0.907 (0.784 - 0.927) |
| **Leadership*** |  |  |
| 1^st^ scenario | 0.923 (0.883 - 0.929) | 0.938 (0.891 - 0.941) |
| 2^nd^ scenario | 0.878 (0.828 - 0.928) | 0.896 (0.859 - 0.917) |
| **Total*** |  |  |
| 1^st^ scenario | 0.867 (0.824 - 0.920) | 0.804 (0.725 - 0.900) |
| 2^nd^ scenario | 0.669 (0.584 - 0.766) | 0.776 (0.763 - 0.785) |

*quadratic weighted kappa for ordinal variables

¶ simple kappa for binary variables
