# Supplementary material for: High-fidelity simulation versus case-based discussion for training undergraduate medical students in pediatric emergencies: a quasi-experimental study
Source: J Pediatr (Rio J). 2024 Apr 9;100(4):422–9. doi: 10.1016/j.jped.2024.03.007 (PMC11331236; doi:10.1016/j.jped.2024.03.007)
Supplement: Supplementary file 7 [file mmc7.docx]

**Table S4**. Descriptive statistics for dependent variables at pre-test (T0) and/or post-test (T1) for intervention and control groups.

| **HFS CBD Total sample** | | | | | | | | | | | | | | |
| --- | --- | --- | --- | --- | --- | --- | --- | --- | --- | --- | --- | --- | --- | --- |
|  |  | **N** | **Mean** | **SD** |  | **N** | **Mean** | **SD** |  | **Min** | **25th** | **50th** | **75th** | **Max** |
| **Self-confidence*** | **T0** | 17 | 59.12 | 22.46 |  | 15 | 50.47 | 14.91 |  | 20,00 | 44,25 | 56,00 | 66.75 | 96.00 |
|  | **T1** | 18 | 93.61 | 23.10 |  | 15 | 88.20 | 18.53 |  | 40,00 | 78.00 | 91.00 | 107.00 | 129.00 |
| **Knowledge **** | **T0** | 16 | 45.05 | 10.23 |  | 14 | 43.45 | 9.20 |  | 29,17 | 37.50 | 41.67 | 52.08 | 66.67 |
|  | **T1** | 18 | 63.19 | 16.86 |  | 15 | 56.67 | 9.29 |  | 37.50 | 50.00 | 58.33 | 66.67 | 100.00 |
| **Simulation checklist***** |  |  |  |  |  |  |  |  |  |  |  |  |  |  |
| **1^st^ scenario** |  |  |  |  |  |  |  |  |  |  |  |  |  |  |
| Total score | **T1** | 18 | 76.21 | 14.60 |  | 15 | 52.13 | 16.46 |  | 14.00 | 53.50 | 68.20 | 81.00 | 93.4 |
| Anamnesis | **T1** | 18 | 64.34 | 11.82 |  | 15 | 50.23 | 9.83 |  | 31.50 | 46.00 | 57.93 | 67.50 | 81.50 |
| Physical examination | **T1** | 18 | 77.64 | 18.90 |  | 15 | 38.87 | 16.29 |  | 14.00 | 43.00 | 57.00 | 85.00 | 100.00 |
| Systematic approach¶ | **T1** | 18 | 16 | 88.89 |  | 15 | 4 | 26.67 |  | - | - | - | - | - |
| Correct diagnosis¶ | **T1** | 18 | 17 | 94.44 |  | 15 | 11 | 73.33 |  | - | - | - | - | - |
| Treatment | **T1** | 18 | 80.11 | 15.48 |  | 15 | 62.77 | 18.89 |  | 17.50 | 59.50 | 74.50 | 85.65 | 100.00 |
| Communication | **T1** | 18 | 79.17 | 22.05 |  | 15 | 57.00 | 26.10 |  | 0.00 | 55.00 | 70.00 | 95.00 | 100.00 |
| Attitude | **T1** | 18 | 80.78 | 21.78 |  | 15 | 55.83 | 35.51 |  | 0.00 | 57.00 | 78.50 | 92.50 | 100.00 |
| Leadership | **T1** | 18 | 84.03 | 24.93 |  | 15 | 60.83 | 34.03 |  | 0.00 | 50.00 | 87.50 | 100.00 | 100.00 |
| **2^nd^ scenario** | | | | | | | | | | | | | | |
| Total score | **T1** | 18 | 68.33 | 11.08 | 15 | | 46.83 | 15.85 | 23.00 | | 48.50 | 62.50 | 72.50 | 87.50 |
| Anamnesis | **T1** | 18 | 50.33 | 14.67 | 15 | | 48.10 | 16.52 | 21.00 | | 37.00 | 53.00 | 57.00 | 81.50 |
| Physical examination | **T1** | 18 | 65.11 | 16.40 | 15 | | 36.13 | 6.11 | 21.00 | | 36.00 | 50.00 | 63.50 | 100.00 |
| Systematic approach¶ | **T1** | 18 | 17 | 94.44 | 15 | | 5 | 33.33 | - | | - | - | - | - |
| Correct diagnosis¶ | **T1** | 18 | 18 | 100 | 15 | | 9 | 60 | - | | - | - | - | - |
| Treatment | **T1** | 18 | 77.42 | 14.13 | 15 | | 57.20 | 18.79 | 22.00 | | 61.00 | 75.00 | 80.00 | 95.50 |
| Communication | **T1** | 18 | 79.17 | 19.04 | 15 | | 49.00 | 31.63 | 0.00 | | 50.00 | 75.00 | 90.00 | 100.00 |
| Attitude | **T1** | 18 | 78.28 | 21.96 | 15 | | 46.83 | 31.67 | 7.00 | | 42.00 | 71.00 | 92.50 | 100.00 |
| Leadership | **T1** | 18 | 81.94 | 25.08 | 15 | | 56.67 | 30.57 | 0.00 | | 50.00 | 87.50 | 100.00 | 100.00 |

CBD – case-based discussion; HSF - high fidelity simulation; SD – standard deviation; T0 – pretest score; T1 – posttest score

*maximum possible score for self-confidence test =144

**maximum possible score for knowledge test =100

***maximum possible total and dimension scores for simulation checklist =100, simulation checklists for two scenarios were applied only at T1 (post-intervention)

¶ results in n and %

All measures had near-normal distributions with kurtosis between 2.344 to 3.749 and skewness between -0.568 and 0.792.
