# Supplementary material for: High-fidelity simulation versus case-based discussion for training undergraduate medical students in pediatric emergencies: a quasi-experimental study
Source: J Pediatr (Rio J). 2024 Apr 9;100(4):422–9. doi: 10.1016/j.jped.2024.03.007 (PMC11331236; doi:10.1016/j.jped.2024.03.007)
Supplement: Supplementary file 8 [file mmc8.docx]

**High-fidelity** **simulation** **versus** **case-based** **discussion** **for** **training** **undergraduate** **medical** **students** **in** **pediatric** **emergencies:** **a** **quasi-experimental** **study.**

Nathalia Veiga Moliterno, Vitor Barreto Paravidino, Jaqueline Rodrigues Robaina, Fernanda Lima-Setta, Antônio José Ledo Alves da Cunha, Arnaldo Prata-Barbosa and Maria Clara de Magalhães-Barbosa.

**Table** **1B.** Percentage distribution of responses to the self-confidence questionnaire in the simulation and discussion groups at baseline.

**Characteristics** **of** **Students**

**Age**

Mean (SD) Median (IQ)

**Gender** Female Male

**Class** **Rating** **(quartiles)** [3-24]

]24-52] ]52-75] ]75-88]

**Simulation** **N** **=17**

24 (1.37) 23.5 (23-25)

**n** **%** **(CI** **95%)**

11 61.1 (38.6-79.7) 7 38.9 (20.3-61.4)

5 27.8 (12.5-50-9) 5 27.8 (12.5-50-9) 5 27.8 (12.5-50-9) 3 16.7 (5.8-39.2)

**Discussion** **N** **=** **15**

24.06 (2.31) 24 (22-25)

**n** **%** **(CI** **95%)**

9 60.0 (35.7-80.2) 6 40.0 (19.8-64.3)

4 26.7 (10.9-52.0) 3 20.0 (7.0-45.2) 3 20.0 7.0-45.2) 5 33.3 (15.2-58.3)

**p-value**

*0.9227a* *0.6164b*

*1.000c*

*0.7922d*

**Distribution** **of** **responses** **to** **the** **Self-confidence** **Questionnaire** **in** **the** **pre-intervention** **phase** **c**

**collect** **an** **adequate** **targeted** **anamnesis** **from** **a** **paediatric** **critical** **patient** 0 lack of trust - -

1 4 22.2 (9.0-45.2) 5 33.3 (15.2-58.3)

2 7 38.9 (20.3-61.4) 8 53.3 (30.1-75.2) *0.466* 3 5 27.8 (12.5-50.9) 1 6.7 (0.3-29.8)

4 full confidence 1 5.6 (0.3-25.8) 1 6.7 (0.3-29.8) **communicate** **clearly** **with** **relatives** **of** **a** **paediatric** **critical** **patient**

0 lack of trust - -

1 2 11.1 (3.10-32.8) 5 33.3 (15.2-58.3)

2 7 38.9 (20.3-61.4) 4 26.7 (10.9-52.0) *0.412* 3 5 27.8 (12.5-50.9) 5 33.3 (15.2-58.3)

4 full confidence 3 16.7 (5.8-39.2) 1 6.7 (0.3-29.8) **welcome** **the** **patient** **and** **family** **during** **paediatric** **emergency** **care**

0 lack of trust - -1 1 5.6 (0.3-25.8)

2 8 44.4 (24.6-66.3) 7 46.7 (24.8-69.9) *0.6537* 3 5 27.8 (12.5-50.9) 7 46.7 (24.8-69.9)

4 full confidence 3 16.7 (5.8-39.2) 1 6.7 (0.3-29.8)

**carry** **out** **clear** **communication** **in** **a** **closed** **loop** **with** **the** **assistance** **team** **during** **emergency** **care** 0 lack of trust 1 5.6 (0.3-25.8) -

1 4 22.2 (9.0-45.2) 6 40.0 (19.8-64.3)

2 7 38.9 (20.3-61.4) 6 40.0 (19.8-64.3) *0.893* 3 4 22.2 (9.0-45.2) 3 20.0 (7.0-45.2)

4 full confidence 1 5.6 (0.3-25.8) **carry** **out** **teamwork** **effectively** **in** **paediatric** **emergency** **care**

0 lack of trust 1 5.6 (0.3-25.8) 1 6.7 (0.3-29.8) 1 4 22.2 (9.0-45.2) 5 33.3 (15.2-58.3)

2 8 44.4 (24.6-66.3) 7 46.7 (24.8-69.9) *0.9388* 3 3 16.7 (5.8-39.2) 1 6.7 (0.3-29.8)

4 full confidence 1 5.6 (0.3-25.8) 1 6.7 (0.3-29.8) **prioritize** **actions** **in** **a** **pediatric** **emergency** **care**

0 lack of trust 2 11.1 (3.10-32.8) 1 6.7 (0.3-29.8) 1 3 16.7 (5.8-39.2) 7 46.7 (24.8-69.9)

2 7 38.9 (20.3-61.4) 5 33.3 (15.2-58.3) *0.4825* 3 4 22.2 (9.0-45.2) 2 13.3 (3.7-37.9)

4 full confidence 1 5.6 (0.3-25.8) -

**Table** **1B** **(cont.).** Percentage distribution of responses to the self-confidence questionnaire in the simulation and discussion groups at baseline.

**identify** **the** **actions** **related** **to** **each** **ABCDE** **approach** **phase**

0 lack of trust 2 11.1 (3.10-32.8) -

1 3 16.7 (5.8-39.2) 3 20.0 (7.0-45.2)

2 7 38.9 (20.3-61.4) 6 40.0 (19.8-64.3) *0.5967* 3 4 22.2 (9.0-45.2) 5 33.3 (15.2-58.3)

4 full confidence 1 5.6 (0.3-25.8) 1 6.7 (0.3-29.8) **prescribe** **medications** **of** **common** **use** **in** **pediatrics** **correctly** **(indication,** **route,** **and** **dose)** 0 lack of trust 6 33.3 (16.3-56.3) 11 73.3 (48.0-89.1)

1 8 44.4 (24.6-66.3) 4 26.7 (10.9-52.0)

2 3 16.7 (5.8-39.2) - *0.06031* 3 - -

4 full confidence - -**recognize** **signs** **and** **symptoms** **of** **pediatric** **respiratory** **failure** 0 lack of trust - -

1 4 22.2 (9.0-45.2) 3 20.0 (7.0-45.2)

2 2 11.1 (3.10-32.8) 6 40.0 (19.8-64.3) *0.2713* 3 10 55.6 (33.7-75.4) 5 33.3 (15.2-58.3)

4 full confidence 1 5.6 (0.3-25.8) 1 6.7 (0.3-29.8) **make** **an** **appropriate** **choice** **in** **the** **type** **of** **device** **for** **oxygen** **therapy** **in** **childhood**

0 lack of trust 5 27.8 (12.5-50.9) 4 26.7 (10.9-52.0) 1 6 33.3 (16.3-56.3) 7 46.7 (24.8-69.9)

2 5 27.8 (12.5-50.9) 3 20.0 (7.0-45.2) *0.893* 3 1 5.6 (0.3-25.8) -

4 full confidence - 27.8 (12.5-50.9) 1 6.7 (0.3-29.8) **intervene** **appropriately** **in** **a** **respiratory** **failure** **in** **a** **child**

0 lack of trust 4 22.2 (9.0-45.2) 4 26.7 (10.9-52.0) 1 7 38.9 (20.3-61.4) 10 66.7 (41.7-84.8)

2 5 27.8 (12.5-50.9) 1 6.7 (0.3-29.8) *0.2724* 3 1 5.6 (0.3-25.8)

4 full confidence -

**assess** **the** **effectiveness** **of** **your** **interventions** **in** **addressing** **respiratory** **failure**

0 lack of trust 3 16.7 (5.8-39.2) 4 26.7 (10.9-52.0) 1 6 33.3 (16.3-56.3) 6 40.0 (19.8-64.3)

2 6 33.3 (16.3-56.3) 4 26.7 (10.9-52.0) *0.8827* 3 2 11.1 (3.10-32.8) 1 6.7 (0.3-29.8)

4 full confidence - -**perform** **proper** **treatment** **of** **a** **child** **with** **upper** **airway** **obstruction?**

0 lack of trust 5 27.8 (12.5-50.9) 3 20.0 (7.0-45.2) 1 5 27.8 (12.5-50.9) 9 60.0 (35.7-80.2)

2 4 22.2 (9.0-45.2) 3 20.0 (7.0-45.2) *0.3732* 3 2 11.1 (3.10-32.8) -

4 full confidence 1 5.6 (0.3-25.8) -**perform** **proper** **treatment** **of** **a** **child** **with** **lower** **airway** **obstruction?**

0 lack of trust 5 27.8 (12.5-50.9) 7 46.7 (24.8-69.9) 1 8 44.4 (24.6-66.3) 6 40.0 (19.8-64.3)

2 3 16.7 (5.8-39.2) 2 13.3 (3.7-37.9) *0.7498* 3 1 5.6 (0.3-25.8) -

4 full confidence

**perform** **adequate** **clinical** **management** **of** **severe** **asthma** **in** **pediatrics** **in** **the** **emergency** **room** 0 lack of trust 4 22.2 (9.0-45.2) 3 20.0 (7.0-45.2)

1 9 50.0 (29.0-71.0) 9 60.0 (35.7-80.2)

2 3 16.7 (5.8-39.2) 3 20.0 (7.0-45.2) *1* 3 1 5.6 (0.3-25.8) -

4 full confidence **-** -

**Table** **1B** **(cont.).** Percentage distribution of responses to the self-confidence questionnaire in the simulation and discussion groups at baseline.

**recognize** **signs** **and** **symptoms** **of** **shock** **in** **pediatrics**

0 lack of trust 2 11.1 (3.10-32.8) 1 6.7 (0.3-29.8) 1 3 16.7 (5.8-39.2) 5 33.3 (15.2-58.3)

2 9 50.0 (29.0-71.0) 7 46.7 (24.8-69.9) *0.7909* 3 3 16.7 (5.8-39.2) 2 13.3 (3.7-37.9)

4 full confidence - -**classify** **shock** **in** **pediatrics** **as** **to** **its** **etiology**

0 lack of trust 5 27.8 (12.5-50.9) 3 20.0 (7.0-45.2) 1 5 27.8 (12.5-50.9) 7 46.7 (24.8-69.9)

2 5 27.8 (12.5-50.9) 5 33.3 (15.2-58.3) *0.5713* 3 2 11.1 (3.10-32.8) -

4 full confidence - -**make** **an** **appropriate** **intervention** **in** **a** **pediatrics** **shock**

0 lack of trust 5 27.8 (12.5-50.9) 5 33.3 (15.2-58.3) 1 7 38.9 (20.3-61.4) 6 40.0 (19.8-64.3)

2 4 22.2 (9.0-45.2) 4 26.7 (10.9-52.0) *1* 3 1 5.6 (0.3-25.8) -

4 full confidence - -**adequately** **approach** **the** **first** **hour** **of** **septic** **shock** **in** **a** **pediatric** **patient**

0 lack of trust 6 33.3 (16.3-56.3) 5 33.3 (15.2-58.3) 1 8 44.4 (24.6-66.3) 9 60.0 (35.7-80.2)

2 3 16.7 (5.8-39.2) 1 6.7 (0.3-29.8) *0.7005* 3 - -

4 full confidence - -

**evaluate** **the** **effectiveness** **of** **your** **interventions** **in** **the** **initial** **approach** **to** **shock** **in** **pediatrics** 0 lack of trust 3 16.7 (5.8-39.2) 5 33.3 (15.2-58.3)

1 8 44.4 (24.6-66.3) 8 53.3 (30.1-75.2)

2 5 27.8 (12.5-50.9) 2 13.3 (3.7-37.9) *0.4838* 3 1 5.6 (0.3-25.8) -

4 full confidence - -

**recognize** **signs** **and** **symptoms** **of** **an** **acute** **neurological** **event** **in** **pediatrics**

0 lack of trust 2 11.1 (3.10-32.8) 4 26.7 (10.9-52.0) 1 4 22.2 (9.0-45.2) 7 46.7 (24.8-69.9)

2 10 55.6 (33.7-75.4) 4 26.7 (10.9-52.0) *0.1472* 3 1 5.6 (0.3-25.8) -

4 full confidence - -**Intervene** **appropriately** **with** **a** **child** **with** **a** **seizure**

0 lack of trust 4 22.2 (9.0-45.2) 4 26.7 (10.9-52.0) 1 5 27.8 (12.5-50.9) 4 26.7 (10.9-52.0)

2 6 33.3 (16.3-56.3) 7 46.7 (24.8-69.9) *0.7244* 3 2 11.1 (3.10-32.8) -

4 full confidence - -**identify** **risk** **factors** **for** **neonatal** **hypoglycaemia**

0 lack of trust - -

1 4 22.2 (9.0-45.2) 6 40.0 (19.8-64.3)

2 10 55.6 (33.7-75.4) 7 46.7 (24.8-69.9) *0.6246* 3 3 16.7 (5.8-39.2) 2 13.3 (3.7-37.9)

4 full confidence - -

**recognise** **signs** **and** **symptoms** **of** **a** **newborn** **with** **neonatal** **hypoglycaemia**

0 lack of trust - 2 13.3 (3.7-37.9) 1 4 22.2 (9.0-45.2) 4 26.7 (10.9-52.0)

2 10 55.6 (33.7-75.4) 7 46.7 (24.8-69.9) *0.6422* 3 3 16.7 (5.8-39.2) 2 3.3 (3.7-37.9)

4 full confidence - -

**Table** **1B** **(cont.).** Percentage distribution of responses to the self-confidence questionnaire in the simulation and discussion groups at baseline.

**intervene** **appropriately** **in** **a** **newborn** **with** **neonatal** **hypoglycemia**

0 lack of trust 2 11.1 (3.10-32.8) 5 33.3 (15.2-58.3) 1 7 38.9 (20.3-61.4) 5 33.3 (15.2-58.3)

2 8 44.4 (24.6-66.3) 5 33.3 (15.2-58.3) *0.3958* 3 - -

4 full confidence - -

**recognise** **signs** **and** **symptoms** **of** **symptomatic** **hypoglycaemia** **in** **a** **child/schoolchild** 0 lack of trust - 3 20.0 (7.0-45.2) 1 5 27.8 (12.5-50.9) 5 33.3 (15.2-58.3)

2 10 55.6 (33.7-75.4) 5 33.3 (15.2-58.3) *0.249* 3 2 11.1 (3.10-32.8) 2 13.3 (3.7-37.9)

4 full confidence - -

**intervene** **appropriately** **in** **a** **child/schoolchild** **with** **symptomatic** **hypoglycaemia**

0 lack of trust 3 16.7 (5.8-39.2) 1 6.7 (0.3-29.8) 1 6 33.3 (16.3-56.3) 8 53.3 (30.1-75.2)

2 7 38.9 (20.3-61.4) 6 40.0 (19.8-64.3) *0.6571* 3 1 5.6 (0.3-25.8) -

4 full confidence - -**diagnose** **patients** **involved** **in** **a** **poisonous** **animal** **accident**

0 lack of trust 1 5.6 (0.3-25.8) 5 33.3 (15.2-58.3) 1 6 33.3 (16.3-56.3) 3 20.0 (7.0-45.2)

2 6 33.3 (16.3-56.3) 6 40.0 (19.8-64.3) *0.1722* 3 4 22.2 (9.0-45.2) 1 6.7 (0.3-29.8)

4 full confidence - -

**classify** **as** **to** **the** **severity** **of** **the** **child** **who** **suffered** **an** **ophidian** **accident**

0 lack of trust 5 27.8 (12.5-50.9) 6 40.0 (19.8-64.3) 1 4 22.2 (9.0-45.2) 6 40.0 (19.8-64.3)

2 6 33.3 (16.3-56.3) 3 20.0 (7.0-45.2) *0.5604* 3 1 5.6 (0.3-25.8) -

4 full confidence 1 5.6 (0.3-25.8) -

**make** **the** **initial** **addressing** **of** **a** **child** **with** **ophidian** **accident** **clinical** **signs**

0 lack of trust 4 22.2 (9.0-45.2) 6 40.0 (19.8-64.3) 1 9 50.0 (29.0-71.0) 8 53.3 (30.1-75.2)

2 2 11.1 (3.10-32.8) 1 6.7 (0.3-29.8) *0.8701* 3 1 5.6 (0.3-25.8) -

4 full confidence 1 5.6 (0.3-25.8) -**recognize** **signs** **and** **symptoms** **of** **anaphylaxis** **in** **pediatrics**

0 lack of trust 1 5.6 (0.3-25.8) 1 6.7 (0.3-29.8) 1 5 27.8 (12.5-50.9) 2 13.3 (3.7-37.9)

2 7 38.9 (20.3-61.4) 10 66.7 (41.7-84.8) *0.5276* 3 4 22.2 (9.0-45.2) 2 13.3 (3.7-37.9)

4 full confidence - -**perform** **adequate** **treatment** **of** **anaphylaxis** **in** **pediatrics**

0 lack of trust 4 22.2 (9.0-45.2) 1 6.7 (0.3-29.8) 1 6 33.3 (16.3-56.3) 9 60.0 (35.7-80.2)

2 6 33.3 (16.3-56.3) 4 26.7 (10.9-52.0) *0.4907* 3 1 5.6 (0.3-25.8) 1 6.7 (0.3-29.8)

4 full confidence - -

**recognize** **signs** **and** **symptoms** **of** **acute** **bacterial** **meningitis** **in** **pediatrics**

0 lack of trust - 1 6.7 (0.3-29.8) 1 3 16.7 (5.8-39.2) 3 20.0 (7.0-45.2)

2 10 55.6 (33.7-75.4) 7 46.7 (24.8-69.9) *0.9398* 3 4 22.2 (9.0-45.2) 4 26.7 (10.9-52.0)

4 full confidence - -

**Table** **1B** **(cont.).** Percentage distribution of responses to the self-confidence questionnaire in the simulation and discussion groups at baseline.

**perform** **appropriate** **diagnostic** **and** **therapeutic** **approach** **of** **pediatric** **patient** **with** **acute** **meningitis**

0 lack of trust 3 16.7 (5.8-39.2) 2 13.3 (3.7-37.9) 1 8 44.4 (0.3-25.8) 10 6.7 (0.3-29.8)

2 6 33.3 (16.3-56.3) 3 20.0 (7.0-45.2) *0.5537* 3 - -

4 full confidence - -**recognize** **signs** **of** **severe** **condition** **in** **an** **adult** **patient**

0 lack of trust - **-**

1 2 11.1 (3.10-32.8) 1 6.7 (0.3-29.8)

2 3 16.7 (5.8-39.2) 4 26.7 (10.9-52.0) *0.9459* 3 10 55.6 (33.7-75.4) 8 53.3 (30.1-75.2)

4 full confidence 2 11.1 (3.10-32.8) 2 13.3 (3.7-37.9) **make** **the** **initial** **approach** **of** **an** **acutely** **ill** **adult** **patient**

0 lack of trust 1 5.6 (0.3-25.8) 1 6.7 (0.3-29.8) 1 2 11.1 (3.10-32.8) 1 6.7 (0.3-29.8)

2 6 38.9 (20.3-61.4) 8 53.3 (30.1-75.2) *0.8499* 3 7 38.9 (20.3-61.4) 5 33.3 (15.2-58.3)

4 full confidence 1 5.6 (0.3-25.8) -a Student's t-test; b Wilcoxon test; c chi-square test; d Fisher test
