# Supplementary material for: High-fidelity simulation versus case-based discussion for training undergraduate medical students in pediatric emergencies: a quasi-experimental study
Source: J Pediatr (Rio J). 2024 Apr 9;100(4):422–9. doi: 10.1016/j.jped.2024.03.007 (PMC11331236; doi:10.1016/j.jped.2024.03.007)
Supplement: Supplementary file 9 [file mmc9.docx]

**High-fidelity** **simulation** **versus** **case-based** **discussion** **for** **training** **undergraduate** **medical** **students** **in** **pediatric** **emergencies:** **a** **quasi-experimental** **study.**

Nathalia Veiga Moliterno, Vitor Barreto Paravidino, Jaqueline Rodrigues Robaina, Fernanda Lima-Setta, Antônio José Ledo Alves da Cunha, Arnaldo Prata-Barbosa and Maria Clara de Magalhães-Barbosa.

**Table** **2B.** Percentage distribution of responses to the multiple-choice theoretical test in the simulation and discussion groups at baseline

**Characteristics** **of** **Students**

**Age**

Mean (SD) 24 Median (IQ) 23.5

**n** **Gender**

Female 11 Male 7 **Class** **Rating**

**(quartiles)**

(3 rd, 24 th] 5 (24º,52º) 5 (52 °,75 °) 5 (75º,88º) 3

**Simulation** **N=18**

(1.37) 24.06 (23-25) 24

**%** **(CI** **95%)** **n**

61.1 (38.6-79.7) 9 38.9 (20.3-61.4) 6

27.8 (12.5-50-9) 4 27.8 (12.5-50-9) 3 27.8 (12.5-50-9) 3 16.7 (5.8-39.2) 5

**Discussion** **N** **=** **15**

(2.31) 22-25

**%** **(CI** **95%)**

60.0 (35.7-80.2) 40.0 (19.8-64.3)

26.7 (10.9-52.0) 20.0 (7.0-45.2) 20.0 (7.0-45.2) 33.3 (15.2-58.3)

**p-value**

*0.9227*

*1,000*

*7,922*

**Distribution** **of** **answers** **to** **the** **theoretical** **Test** **Questionnaire** **in** **the** **pre-intervention** **phase**

**In** **pediatric** **emergency** **situations,** **facing** **an** **unconscious** **(unresponsive)** **infant,** **which** **of** **the** **pulses** **described** **below** **are** **the** **ones** **of** **choice** **for** **verification** **if** **the** **patient** **is** **in** **cardiac** **arrest?** 0 9 56.3 (33.2-76.9) 4 28.6 (11.7-54.6) *0,1590* 1 7 43.8 (23.1- 66.8) 10 71.4 (45.4- 88.3)

**2.** **In** **pediatric** **emergency** **situations,** **facing** **an** **unconscious** **(unresponsive)** **student,** **which** **of** **the** **two** **pulses** **described** **below** **are** **the** **one** **of** **choice** **for** **verification**

0 8 50.0 (28.0-72.0) 7 50.0 (26.8- 73.2) *1,000* 1 8 50.0 (28.0-72.0) 7 50.0 (26.8- 73.2)

**In** **pediatric** **patients,** **the** **main** **cause** **of** **cardiac** **arrest** **is** **due** **to:** 0 6 37.5 (18.5- 61.4) 9

1 10 62.5 (38.6-81.51) 5

64.3 (38.8- 83.7) *0.2723* 35.7 (16.3-61.2)

**4A** **20kg** **child** **has** **fever,** **irritability,** **cold** **extremities** **and** **prolonged** **hair** **filling** **time.** **The** **appropriate** **initial** **fluid** **for** **bolus** **administration** **for** **this** **child** **is:**

0 4 25.0 (10.2-49.5) 7 50.0 (26.8- 73.2) *0.2568* 1 12 75.0 (50.5-89.8) 7 50.0 (26.8- 73.2)

**A** **3-year-old** **child** **arrives** **at** **the** **emergency** **room** **with** **a** **low** **level** **of** **consciousness,** **pale,** **central** **cyanosis** **1+/4+,** **thin** **wrists,** **moderate** **respiratory** **effort.** **On** **the** **basis** **of** **this** **initial** **situation,** **its** **first** **intervention** **should** **be:**

0 4 25.0 (10.2-49.5) 4 28.6 (11.7-54.6) *1,000* 1 12 75.0 (50.5-89.8) 10 71.4 (45.4- 88.3)

**The** **inhaled** **dose** **of** **epinephrine** **for** **infants** **or** **children** **is:** 0 15 93.8 (71.7-99.7) 12 1 1 6.3 (0.3-28.3) 2

85.7 (60.1-96.0) *0.5862* 14.3 (4.0- 39.9)

**A** **previously** **healthy** **5-month-old** **infant** **was** **admitted** **to** **the** **cyanotic** **emergency** **room,** **with** **red** **spots** **throughout** **the** **body,** **irritability,** **and** **respiratory** **distress** **beginning** **in** **30** **minutes.** **The** **grandmother** **who** **brought** **the** **child.** **She** **claims** **to** **have** **offered** **infant** **formula** **today** **for** **the** **first** **time.** **|||UNTRANSLATED_CONTENT_START|||No** **exame** **físico** **de** **admissão:** **Agitado,** **pálido,** **manchas** **pelo** **corpo,** **PCP** **lentificada,** **pulsos** **periféricos** **finos** **e** **rápidos.** **|||UNTRANSLATED_CONTENT_END|||Tachypneic,** **moderate** **respiratory** **effort,** **MVUA,** **bilateral** **wheezing.** **Erythematous** **papules** **on** **the** **trunk,** **face** **and** **limbs.** **Absence** **of** **meningeal** **signs.** **HR** **195** **FR:** **50** **SatO2:** **88%** **Tax** **36°C** **PA** **90X55mmHg** **(normal** **for** **age).** **Given** **the** **situation** **presented,** **what** **is** **the** **primary** **conduct** **for** **solving** **the** **case?**

0 3 18.8 (6.6- 43.0) 5 35.7 (16.3-61.2) *4171* 1 13 81.3 (56.9-93.4) 9 64.3 (38.8- 83.7)

**Table** **2B** **(cont.).** Percentage distribution of responses to the multiple-choice theoretical test in the simulation and discussion groups at baseline.

**The** **inhaled** **dose** **of** **epinephrine** **for** **infants** **or** **children** **is:** 0 4 25.0 (10.2-49.5) 3

1 12 75.0 (50.5-89.8) 11

21.4 (7.6-47.6) *1,000* 78.6 (52.4-92.4)

**A** **15-month-old** **infant,** **weighing** **13** **kg,** **presented** **hyaline** **coryza,** **dry** **cough** **and** **fever** **of** **38ºC** **this** **evening.** **He** **was** **treated** **with** **paracetamol,** **with** **a** **good** **response,** **and** **fell** **asleep.** **After** **4** **hours** **of** **sleep,** **he** **started** **an** **intense** **episode** **of** **barking** **cough,** **hoarseness** **and** **progressive** **dyspnea.** **On** **examination,** **he** **presented** **stridor** **at** **rest,** **cyanosis** **of** **the** **extremities** **and** **subcostal** **circulation.** **His** **parents** **immediately** **took** **him** **to** **the** **Emergency** **Care** **Unit.** **What** **specific** **therapeutic** **conduct** **should** **be** **initiated** **in** **this** **case?**

0 13 81.3 (56.9-93.4) 13 92.9 (68.5 99.6) *0.6015* 1 3 18.8 (6.6- 43.0) 1 7.1 (0.4-31.5)

**The** **first** **clinical** **manifestation** **of** **shock** **in** **pediatrics** **is:**

0 3 18.8 (6.6- 43.0) 4 28.6 (11.7-54.6) *6746* 1 13 81.3 (56.9-93.4) 10 71.4 (45.4- 88.3)

**Among** **the** **situations** **below,** **in** **which** **of** **them** **could** **you** **perform** **lumbar** **puncture**?

0 12 75.0 (50.5-89.8) 9 64.3 (38.8- 83.7) *0.6944* 1 4 25.0 (10.2-49.5) 5 35.7 (16.3-61.2)

**A** **10-year-old** **student** **weighing** **35** **kg,** **diagnosed** **with** **diabetes** **mellitus** **for** **10** **months,** **was** **on** **NPH** **insulin** **in** **the** **morning** **and** **in** **the** **evening** **before** **meals.** **He** **arrived** **at** **the** **emergency** **room** **at** **10:** **00** **a.m.** **with** **a** **history** **of** **fainting,** **sweating** **and** **tremors** **at** **school.** **When** **asked** **by** **the** **emergency** **physician,** **the** **mother** **mentioned** **that** **the** **minor** **did** **not** **want** **to** **have** **coffee** **before** **going** **to** **school.** **Intake** **capillary** **blood** **glucose** **30** **mg/dl.** **What** **is** **the** **correct** **treatment** **for** **this** **clinical** **situation?**

0 14 87.5 (64.0-96.5) 14 100.0 (78.5-100.0) *0.4851* 1 2 12.5 (3.5- 36.0) 0 0.0 (0.0- 21.5)

**A** **15-month-old** **infant** **was** **admitted** **to** **the** **Emergency** **Care** **Unit** **unconscious** **presenting** **conjugate** **deviation** **of** **the** **look** **up,** **spasticity** **of** **the** **limbs** **and** **sialorrhea.** **The** **mother** **reports** **that** **the** **painting** **started** **20** **minutes** **ago.** **Estimated** **weight** **of** **10** **kg.** **After** **the** **initial** **critical** **patient** **approach,** **which** **of** **the** **following** **indicates** **the** **correct** **conduct** **to** **be** **initiated?**

0 12 75.0 (50.5-89.8) 12 85.7 (60.1-96.0) *0.6567* 1 4 25.0 (10.2-49.5) 2 14.3 (4.0- 39.9)

**14.** **The** **results** **found** **in** **cerebrospinal** **fluid** **that** **suggest** **bacterial** **meningitis** **are:**

0 1 6.3 (0.3-28.3) 3 21.4 (7.6-47.6) *3155* 1 15 93.8 (71.7-99.7) 11 78.6 (52.4-92.4)

**Faced** **with** **a** **preschooler** **with** **decompensated** **shock,** **what** **is** **the** **vascular** **access** **of** **choice** **for** **infusion** **of** **fluid** **therapy?**

0 11 68.8 (44.4-85.8) 9 64.3 (38.8- 83.7) *1,000* 1 5 31.3 (14.2- 55.6) 5 35.7 (16.3-61.2)

**The** **intervention** **that** **has** **a** **direct** **effect** **on** **reducing** **mortality** **in** **anaphylaxis** **is:**

0 4 25.0 (10.2-49.5) 2 14.3 (4.0- 39.9) *0.6567* 1 12 75.0 (50.5-89.8) 12 85.7 (60.1-96.0)

**NB** **with** **48** **hours** **of** **life,** **premature** **at** **35** **weeks** **and** **5** **days,** **due** **to** **pre-eclampsia,** **PN** **2450g.** **She** **is** **staying** **with** **her** **exclusively** **breastfed** **mother.** **Mother** **says** **that** **"he** **is** **lazy",** **nurse** **noticed** **that** **he** **is** **presenting** **tremors** **in** **limbs** **and** **sweating** **and** **immediately** **called** **the** **pediatrician** **on** **duty,** **who** **performed** **capillary** **blood** **glucose** **(23** **mg/dl).** **What** **is** **the** **most** **appropriate** **conduct?**

0 12 75.0 (50.5-89.8) 13 92.9 (68.5 99.6) *3359* 1 4 25.0 (10.2-49.5) 1 7.1 (0.4-31.5)

**A** **4-year-old** **child** **was** **admitted** **to** **the** **emergency** **room** **with** **coughing,** **tiredness** **and** **“wheezing** **in** **the** **chest”.** **On** **examination,** **the** **patient** **was** **found** **to** **have** **dyspnea** **with** **a** **notch** **and** **a** **wing** **beat** **of** **the** **nose,** **Sato2** **88%,** **respiratory** **auscultation** **with** **diffuse** **wheezing.** **The** **treatment** **sequence** **after** **initiation** **of** **inhaled** **oxygen** **therapy** **should** **be:**

0 7 43.8 (23.1- 66.8) 2 14.3 (4.0- 39.9) *0.1184* 1 9 56.3 (33.2-76.9) 12 85.7 (60.1-96.0)

**Table** **2B** **(cont.).** Percentage distribution of responses to the multiple-choice theoretical test in the simulation and discussion groups at baseline.

**What** **is** **the** **maximum** **dose** **of** **adrenaline** **and** **minimum** **interval** **for** **subsequent** **dose,** **if** **necessary,** **for** **treatment** **of** **anaphylactic** **shock?**

0 12 75.0 (50.5-89.8) 10 71.4 (45.4- 88.3) *1,000* 1 4 25.0 (10.2-49.5) 4 28.6 (11.7-54.6)

**Among** **the** **actions** **that** **are** **a** **priority** **in** **the** **approach** **of** **pediatric** **patients** **in** **the** **first** **hour** **of** **septic** **shock** **are,** **except:**

0 12 75.0 (50.5-89.8) 8 57.1 (32.6-78.6) *4421* 1 4 25.0 (10.2-49.5) 6 42.9 (21.4-67.4)

**A** **9-month-old** **male** **patient** **was** **admitted** **to** **the** **emergency** **room** **with** **diffuse** **wheezing** **and** **moderate** **respiratory** **distress** **(tachypnea** **and** **subcostal** **circulation).** **Mother** **reports** **recurrent** **episodes** **of** **wheezing** **associated** **with** **“viruses”.** **In** **the** **last** **three** **months** **it** **was** **three** **times** **the** **emergency** **for** **the** **same** **reason.** **Weight** **10kg** **-** **Sato2** **90%.** **The** **medication** **and** **dose** **indicated** **for** **rescue:**

0 4 25.0 (10.2-49.5) 7 50.0 (26.8- 73.2) *0.2568* 1 12 75.0 (50.5-89.8) 7 50.0 (26.8- 73.2)

**In** **cases** **of** **ophidian** **accident,** **we** **base** **the** **calculation** **of** **the** **amount** **of** **ampoules** **of** **antivenom** **to** **be** **administered:**

0 15 93.8 (71.7-99.7) 13 92.9 (68.5 99.6) *1,000* 1 1 6.3 (0.3-28.3) 1 7.1 (0.4-31.5)

**In** **cases** **of** **botropic** **accident,** **we** **must** **NOT:**

0 10 62.5 (38.6-81.51) 6 1 6 37.5 (18.5- 61.4) 8

57.1 (32.6-78.6) *0.4642* 42.9 (21.4-67.4)

**A** **4-year-old** **boy** **underwent** **lumbar** **puncture** **and** **the** **cerebrospinal** **fluid** **results** **showed** **a** **cloudy** **cerebrospinal** **fluid,** **cellularity** **of** **1000** **cells/mm3** **with** **a** **predominance** **of** **polymorphonuclear** **cells** **(90%);** **proteinorrhachia** **of** **250mg/dL,** **glycorrhachia** **of** **23mg/dL** **(glycemia** **80mg/dL)** **.** **Bacterioscopy** **showed** **numerous** **Gram** **positive** **coconuts** **in** **pairs** **andchains.What**

**is** **the** **most** **likely** **etiologic** **agent** **for** **meningitis** **described** **above?**

0 16 100.0 (80.6- 100.0) 14 100.0 (78.5-100.0) *-*1 0 0.0 (0.0-19.4) 0 0.0 (0.0- 21.5)
