# Supplementary material for: High-fidelity simulation versus case-based discussion for training undergraduate medical students in pediatric emergencies: a quasi-experimental study
Source: J Pediatr (Rio J). 2024 Apr 9;100(4):422–9. doi: 10.1016/j.jped.2024.03.007 (PMC11331236; doi:10.1016/j.jped.2024.03.007)
Supplement: Supplementary file 10 [file mmc10.docx]

Supplement 6

**Table 6.1**. Results from mixed-effect models time x group analysis of effects on pre-to post intervention gains in self-confidence and knowledge outcomes.

| Self-confidence DF *p-value* Knowledge DF *p-value* | | | | | | | |
| --- | --- | --- | --- | --- | --- | --- | --- |
|  |  | Estimate (SE) |  |  | Estimate (SE) |  |  |
| Fixed effects | Intercept | 40.40 (7.81) | 30 | *<.0001* | 50.34 (4.45) | 31 | *<0.0001* |
|  | Time | 37.73 (4.38) | 30 | *<.0001* | 12.98 (3.46) | 27 | *<0.001* |
|  | Group | 9.06 (6.97) | 30 | *0.2035* | 1.23 (4.26) | 27 | *0.7749* |
|  | Ranking | 0.19 (0.11) | 30 | *0.0991* | -0.13 (0.06) | 27 | *0.0506* |
|  | **Time x Group** | -2.69 (5.99) | 30 | ***0.6565*** | 4.66 (4.73) | 27 | ***0.3331*** |
| Hedges’ g | Time x group | -0.112 |  |  | 0.325 |  |  |
| (SE) |  | (0.280) |  |  | (0.282) |  |  |

DF – degrees of freedom; SE – standard error

The group effect (not crossed by time) shows the difference between groups at baseline. This difference was not statistically significant for both the self-confidence and the knowledge measures at baseline. The covariate student ranking marginally predicted self-confidence (p = 0.09) and knowledge (p = 0.05). The time vs. group row represents the critical test of the group on gains from pre to post-test for each measure, and the bottom row in the table shows the effect sizes (Hedges g) for that critical test. No difference between groups was observed regarding changes in the scores of both tests over time (p=0.6565 for the self-confidence test; p=0.3331 for the knowledge test). The time effect was significant for both groups in the self- confidence test (p<000.1 {HFS] and p<0.001 [CDB]) and in the knowledge test (p=0.001 {HFS] and p< 0.01 ([CBD]).

**Table 6.2** – Results from linear and logistic regression models to test group effect on simulation checklist scores

| 1^st^ Scenario (n = 33) | | | | | | | | | | | | |
| --- | --- | --- | --- | --- | --- | --- | --- | --- | --- | --- | --- | --- |
| Predictors | Anamesis^a^ | Physical exam^a^ | Treatment^a^ | Systematic Approach^b^ | Correct diagnosis^b^ | Communication^a^ | Attitude^a^ | | Leadership^a^ | | Total score^a^ | |
|  | Estimate  (95% CI) | Estimate (95% CI) | Estimate  (95% CI) | Estimate  (95% CI) | Estimate (95% CI) | Estimate (95% CI) | Estimate  (95% CI) | | Estimate  (95% CI) | | Estimate (95% CI) | |
| Intercept | 54.64*** | 43.23*** | 77.14*** | 1.89 | 136.84˜ | 60.71*** | 75.80*** | | 87.97*** | | 63.03*** | |
|  | (45.33; 63.95) | (27.92; 58.55) | (63.91; 90.37) | (0.25; 16.33) | (3.61; 333308.98) | (39.94; 81.48) | (52.61; 99.00) | | (65.87; 110.07) | | (50.61; 75.45) | |
| Group | 13.69 * | 38.36*** | 15.97** | 32.30** | 6.59 | 21.81* | 23.04* | | 20.60* | | 23.03*** | |
|  | (5.89; 21.49) | (25.53; 51.19) | (4.88; 27.06) | (4.72; 474.19) | (0.69; 157.33) | (4.41; 39.21) | (3.61; 42.48) | | (2.09; 39.12) | | (12.62; 33.44) | |
| Ranking | -0.09 | -0.08 | -0.28** | 0.96˜  (0.92; 1.00) | 0.94˜  (0.85; 0.99) | -0.07 | -0.39* | | -0.53** | | -0.21* | |
|  | (-0.23; 0.06) | (-0.32; 0.15) | (-0.48; -0.08) |  |  | (-0.39; 0.25) | (-0.74; -0.03) | | (-0.87; -0.19) | | (-0.40; -0.02) | |
| R2/R2 adj. | 0.337 / 0.293 | 0.565 / 0.536 | 0.378 / 0.336 | - | - | 0.190 / 0.136 | 0.284 / 0.236 | | 0.358 / 0.315 | | 0.480 / 0.445 | |
| R2 Tjur | - | - |  | 0.475 | 0.253 | - | - | | - | | - | |
| Hedges’ g | 1.26 (0.37) | 2.13 (0.43) | 0.99 (0.36) | 1.87 (0.57) ‡ | 1.11 (0.71) ‡ | 0.90 (0.12) | 0.94 (0.36) | | 0.77 (0.35) | | 1.52 (0.38) | |
| 2^nd^ Scenario (n=33) | | | | | | | | | | | | |
|  | Anamesis^a^ | Physical exam^a^ | Treatment^a^ | Systematic Approach^b^ | Correct  diagnosis**$** | Communication^a^ | | Attitude^a^ | | Leadership^a^ | | Total score^a^ |
| Predictors | Estimate | Estimate | Estimate | Estimate | Estimate | Estimate | | Estimate | | Estimate | | Estimate |
|  | (95% CI) | (95% CI) | (95% CI) | (95% CI) | (95% CI) | (95% CI) | | (95% CI) | | (95% CI) | | (95% CI) |
| Intercept | 57.53*** | 35.94*** | 67.20*** | 2.53 | - | 60.95*** | | 61.87*** | | 69.00*** | | 55.59*** |
|  | (44.78; 70.28) | (24.79; 47.09) | (53.74; 80.65) | (0.31; 26.25) |  | (39.50; 82.41) | | (39.68; 84.05) | | (45.62; 92.37) | | (44.65; 66.52) |
| Group | 1.33 | 29.00*** | 19.26** | 46.38** | - | 29.03** | | 30.01** | | 24.10** | | 20.66*** |
|  | (-9.35; 12.01) | (19.66; 38.34) | (7.99; 0.53) | (5.54; 1192.04) |  | (11.05; 47.00) | | (11.43; 48.59) | | (4.52; 43.68) | | (11.50; 29.83) |
| Ranking | -0.18 | 0.00 | -0.19 | 0.97˜ | - | -0.23 | | -0.29˜ | | -0.24 | | -0.17* |
|  | (-0.38 – 0.01) | (-0.17; 0.17) | (-0.40; 0.01) | (0.92; 1.00) |  | (-0.56; 0.10) | | (-0.63; 0.05) | | (-0.60; 0.12) | | (-0.34; -0.00) |
| R2/R2 adj. | 0.114 / 0.055 | 0.574 / 0.546 | 0.365 / 0.323 | - |  | 0.317 / 0.272 | | 0.335 / 0.291 | | 0.228 / 0.177 | | 0.478 / 0.443 |
| R2 Tjur | - | - | - | 0.483 | - | - | | - | | - | | - |
| Hedges’ g | 0.14 (0.34) | 2.20 (0.43) | 1.20 (0.37) | 2.14 (0.70) ‡ | - | 1.15 (0.37) | | 1.15 (0.37) | | 0.89 (0.36) | | 1.56 (0.39) |

a linear regression; ^b^ logistic regression

‡ The cox index (dcox) was used to estimate the effect size for dichotomous outcomes, according to the What Works Clearinghouse Procedures Handbook v.4 (WWC, 2014).

∼ p < .10; ∗ p < .05; ∗∗ p < .01; ∗∗∗ p < .001

**$** It was not possible to conduct a logistic regression or estimate the effect size because all students (100%) in the High-Fidelity simulation group got the right diagnosis
